# Natural killer cells and innate lymphoid cells but not NKT cells are mature in their cytokine production at birth

**DOI:** 10.1093/cei/uxad094

**Published:** 2023-08-09

**Authors:** Dawid Swieboda, Thomas F Rice, Yanping Guo, Simon Nadel, Ryan S Thwaites, Peter J M Openshaw, Beth Holder, Fiona J Culley

**Affiliations:** National Heart and Lung Institute, Department of Metabolism, Digestion and Reproduction, Faculty of Medicine, Imperial College London, UK; National Heart and Lung Institute, Department of Metabolism, Digestion and Reproduction, Faculty of Medicine, Imperial College London, UK; National Heart and Lung Institute, Department of Metabolism, Digestion and Reproduction, Faculty of Medicine, Imperial College London, UK; National Heart and Lung Institute, Department of Metabolism, Digestion and Reproduction, Faculty of Medicine, Imperial College London, UK; National Heart and Lung Institute, Department of Metabolism, Digestion and Reproduction, Faculty of Medicine, Imperial College London, UK; National Heart and Lung Institute, Department of Metabolism, Digestion and Reproduction, Faculty of Medicine, Imperial College London, UK; National Heart and Lung Institute, Department of Metabolism, Digestion and Reproduction, Faculty of Medicine, Imperial College London, UK; National Heart and Lung Institute, Department of Metabolism, Digestion and Reproduction, Faculty of Medicine, Imperial College London, UK

**Keywords:** human, natural killer cells, innate lymphoid cells

## Abstract

Early life is a time of increased susceptibility to infectious diseases and development of allergy. Innate lymphocytes are crucial components of the initiation and regulation of immune responses at mucosal surfaces, but functional differences in innate lymphocytes early in life are not fully described. We aimed to characterize the abundance and function of different innate lymphocyte cell populations in cord blood in comparison to that of adults. Blood was collected from adult donors and umbilical vessels at birth. Multicolor flow cytometry panels were used to identify and characterize lymphocyte populations and their capacity to produce hallmark cytokines. Lymphocytes were more abundant in cord blood compared to adults, however, mucosal-associated invariant T cells and natural killer T (NKT)-like cells, were far less abundant. The capacity of NKT-like cells to produce cytokines and their expression of the cytotoxic granule protein granzyme B and the marker of terminal differentiation CD57 were much lower in cord blood than in adults. In contrast, natural killer (NK) cells were as abundant in cord blood as in adults, they could produce IFNγ, and their expression of granzyme B was not significantly different from that of adult NK cells, although CD57 expression was lower. All innate lymphoid cell (ILC) subsets were more abundant in cord blood, and ILC1 and ILC2 were capable of production of IFNγ and IL-13, respectively. In conclusion, different innate lymphoid cells differ in both abundance and function in peripheral blood at birth and with important implications for immunity in early life.

## Teaser

Innate lymphocytes may be important for immunity in early life. Natural killer T- (NKT-) and mucosal-associated invariant T-cells were found in low abundance in cord blood and NKT cells from cord blood were less capable of cytokine and granzyme B production than those from adults. In contrast, cord blood natural killer cells and subsets of innate lymphoid cells were abundant and capable of producing their hallmark cytokines.

## Introduction

Early life is generally a time of increased susceptibility to infections, particularly of mucosal sites such as the gastrointestinal and respiratory tracts. Furthermore, allergic disease often develops in early life. In the context of an immature and inexperienced adaptive immune system, innate immunity and particularly that at mucosal sites may dictate the outcome of allergen and infectious challenge [1–3]. The differences in and development of innate immunity in early life are not fully understood.

Innate lymphocytes are particularly abundant at mucosal sites where they respond rapidly to environmental insults and infection, contribute to homeostasis and integrity of mucosal tissues, and regulate immunity and inflammation [4]. Innate lymphocytes include a diverse array of cells, such as innate lymphoid cells (ILC) and unconventional T lymphocytes expressing invariant T-cell receptors [2, 5]. ILCs have both inflammatory and immunoregulatory functions. They support the maintenance and repair of barrier function in the gut and in the lung [6–8] and have been implicated in inflammatory diseases of barrier sites including asthma, psoriasis, and Crohn’s disease [9]. Unconventional T lymphocytes include mucosal-associated invariant T (MAIT) cells, γδT cells, invariant natural killer T (iNKT), and intraepithelial lymphocytes [10, 11]. These cells can be activated by diverse stimuli including microbial components and stress-induced molecules. For example, MAIT cells recognize microbial intermediates in B vitamin synthesis in the context of the MHC-related 1 (MR1) molecule, as well as being activated by inflammatory stimuli [10, 12–14] and are increasingly recognized as playing roles in homeostasis and infectious diseases [10, 15]. Natural killer T (NKT) cells are CD3^+^ CD56^+^ T lymphocytes. A major subset of NKT cells in humans express a T-cell receptor (TCR) Vα24-Jα18 chain paired with the TCRβ11 chain and respond to glycolipids presented by the MHC-I-like non-polymorphic protein CD1d; however, NKT cells are diverse in their TCR chain usage and antigen recognition [11]. NK cells contribute to protection against infection by both cytotoxicity and cytokine production [16, 17].

ILCs are found in a greater abundance in cord blood and the peripheral blood of infants compared to healthy adults [18] and in early life unconventional innate-like T cells accumulate in tissues and mature under the influence of signals from the newly acquired microbiota [19]. Higher levels of type 2 ILC (ILC2) have been reported in the airways of infants with severe lower respiratory tract infection with respiratory syncytial virus (RSV) [20] and in murine neonatal RSV infection, ILC2 are a source of IL-13 in the lungs [21]. ILC2 are found in elevated numbers in the airways of children with severe therapy-resistant asthma [22, 23]. In murine models, activation of type 3 ILC (ILC3) can drive pathology in the neonatal gut [24]. Together with the ability of innate lymphocytes to influence neonatal adaptive immunity [25], this evidence suggests that innate lymphocytes play important roles in infectious and inflammatory diseases in early life.

A better understanding of the functional differences in innate lymphocytes in neonates may generate insights into early-life immune defense and pathology, particularly at mucosal sites. In this study, we aimed to define functional differences in subsets of innate lymphocytes in adults and neonates. As ILCs can mediate their functions by secretion of cytokines and cytotoxicity, we compared ILC subset frequency and their ability to produce canonical cytokines and the cytotoxic granule component granzyme B. We find striking differences in the functions and frequencies of different innate lymphocyte populations in cord blood and adult blood. MAIT cells and NKT cells were found at low abundance in cord blood, and NKT cells had a low capacity for cytokine production. In contrast, NK cells and ILCs were abundant in cord blood and could produce cytokines at comparable levels to adult peripheral blood cells.

## Methods

### Samples

Peripheral blood from healthy adult volunteers (*n* = 15) or umbilical cord blood (*n* = 17) was collected into EDTA-coated tubes (BD, UK). Adult volunteers had a median age of 32 years (range 23–63). Cord blood was obtained from the *ex utero* placenta from healthy, term, singleton pregnancies delivered by elective cesarean section at St Mary’s Hospital, London, with a median of 39.29 weeks (range 38.43–40.14) of gestation at delivery. There were no significant differences in the birth weight between males and females in the cord blood cohort. The details of samples used for each analysis are shown in Supplementary Table S1. Samples were obtained following informed consent under local research ethics committee approval (REC 15/WM/0343 and 13/LO/1712).

### Enumeration of cell populations in peripheral blood

A 14-color, 17-parameter flow cytometry panel was used to enumerate different cell populations as described [26]. Briefly, fresh whole blood samples were stained for 30 min at room temperature with a panel of antibodies to lymphocyte surface markers (Supplementary Table S2) and 1:500 live/dead fixable near-infrared (near-IR) dye (Thermo Fisher Scientific, UK), followed by fixation and red blood cell lysis using red blood cell lysis/fixation solution (BioLegend, UK). After washing, CountBright™ absolute counting beads (Invitrogen, UK) were added to the sample and data was acquired using an LSR-Fortessa cell analyzer (BD Bioscience). Instrument calibration using cytometry setting and tracking (CST) beads, and compensation using AbC total compensation beads (Thermo Fisher Scientific, UK) and ArC Amine reactive compensation beads (Invitrogen, UK), were performed for each experiment.

### Functional analysis by intracellular staining

Peripheral blood mononuclear cells (PBMC) were obtained by centrifugation of blood samples over a lymphopure (BioLegend, UK) density gradient. Cells obtained from the interface were counted and frozen in 10% dimethylsulphoxide (DMSO), and 90% fetal calf serum (FCS; Sigma-Aldrich, UK). For analysis, PBMC were thawed, washed into complete medium (10% FCS, 100 U/mL penicillin, 100μg/ml streptomycin, 2 mM l-glutamine in RPMI-1640), and left to rest overnight in a humidified incubator at 37°C, 5% CO_2_. For characterization of cytokine production, samples were stimulated for 3 h at 37°C with ionomycin (1 µg/ml) and phorbol 12-myristate 13-acetate (PMA; 50 ng/ml), or left unstimulated (DMSO vehicle only), in the presence of Golgiplug (BD, UK) at a 1:1000 dilution. Cells were next incubated with Fc block (anti-CD16/CD32; Biolegend, UK) for 10 min, followed by a panel of antibodies to surface markers (Supplementary Table S3) and live/dead Fixable Near-IR dye diluted in PBS (1:500), for 30 min at 4°C, then washed, and fixed with fixation buffer (BD, UK). For intracellular staining, cells were washed with permeabilization wash buffer (BioLegend, UK) followed by incubation for 60 min at 4°C with antibodies to cytokines, diluted in permeabilization wash buffer (Supplementary Table S3). Samples were washed and data was acquired using an LSR-Fortessa cell analyzer (BD Bioscience), as above. The intracellular cytokine staining panels were optimized by titration of antibodies on PBMC from adult blood. Data analysis gates were set using fluorescence minus one (FMO) and unstimulated sample controls.

For analysis of CD57 and granzyme B expression, PBMC were incubated overnight and were not stimulated. Cells were stained with antibodies to surface markers, followed by permeabilization and intracellular staining with an antibody to granzyme B, and data acquisition, as above (Supplementary Table S4).

### Data analysis

Data were analyzed and graphs were generated using FlowJo V10.6 software (FlowJo LLC, USA), Microsoft Excel (Microsoft, Washington, USA), GraphPad Prism 9.0 (GraphPad Software, Inc., California, USA), and R studio (Versions 1.2.5033, 2009-2010 and 2023.06.0 + 421, RStudio, Inc). Lognormality was tested using the D’Agostino and Pearson test in GraphPad Prism. As not all data were lognormally distributed, *P* values were calculated by Mann–Whitney test unless otherwise stated. The heatmap was produced in R Studio using the ComplexHeatmap package [27]. For the heatmap, data were log transformed and scaled by subtraction the mean of each population from each value and dividing by the standard deviation. PCA analysis was performed using the FactoMineR package in R Studio.

## Results

### High diversity in blood lymphocyte populations at birth

**Figure 1.**
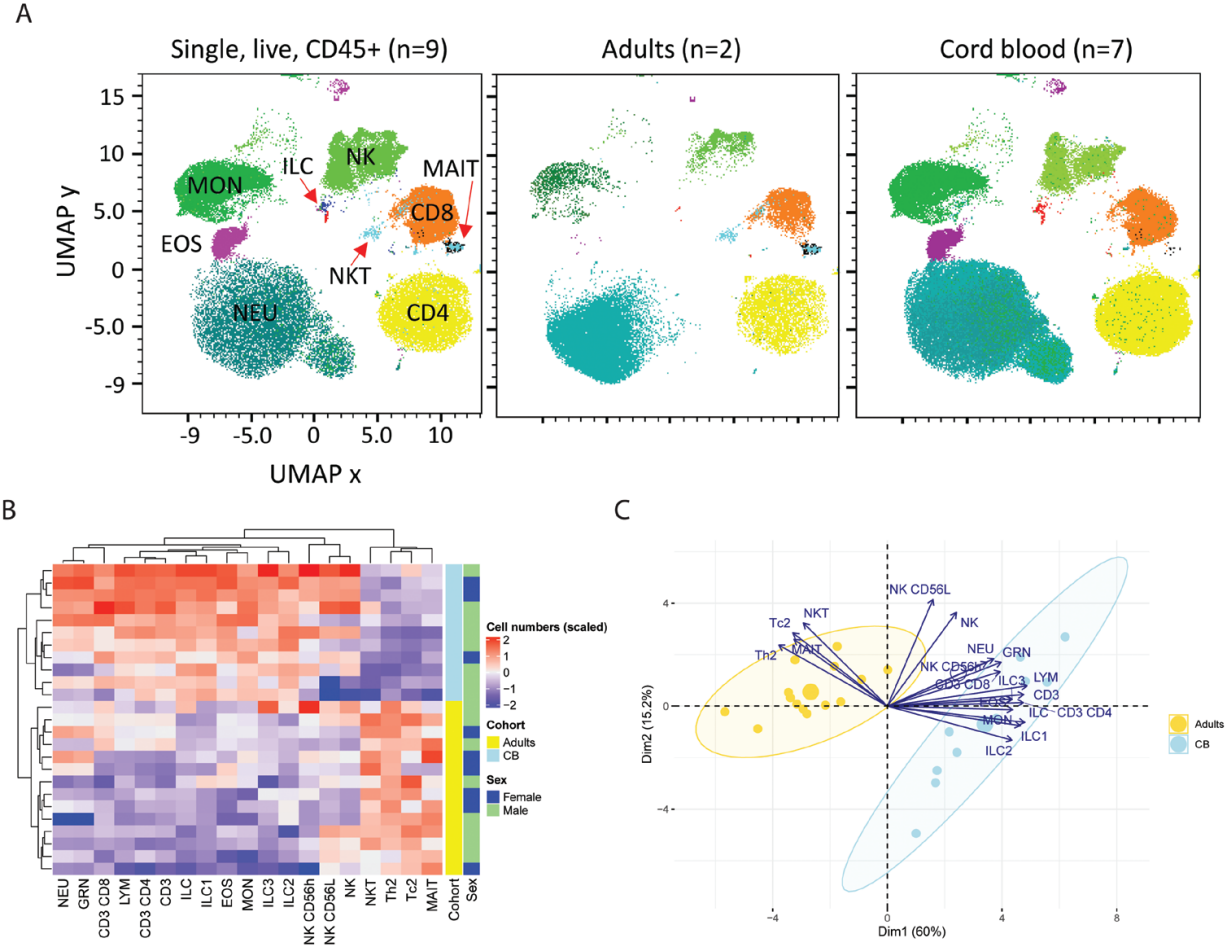
Multidimensional analysis of cell abundance in adult and cord blood. (**A**) UMAP dimensionality reduction was performed and a manual gating strategy used to classify UMAP clusters into the following cell types: CD4 + T cells (CD4; yellow), CD8 + T cells (CD8; orange), NKT cells (NKT; bright blue), monocytes (MON; green), neutrophils (NEU; ocean blue), eosinophils (EOS; magenta), NK cells (NK; bright green), MAIT cells (MAIT; black), ILC3 (purple), ILC2 (blue) and ILC1 (red). The populations of MAIT cells, ILCs and NKT-like cells are indicated via red arrows (left). Comparison of UMAP representations of clusters for adult subjects (*n* = 2, middle) and cord blood (*n* = 7, right). **(B)** Abundance per ml blood of different leukocyte populations displayed in a heatmap where each row represents an individual donor and each column represents a cell population. The rows and columns were ordered according to unsupervised hierarchical clustering, putting similar observations close to each other. Data sets were first scaled for each cell population. High values are in red and low values in blue. The rows are annotated according to cohort (adults, cord blood (CB)) and sex (female, male). **(C)** Principal component analysis (PCA), where each individual is plotted as a point and individuals that are similar are grouped together, using cell population abundance (numbers per ml blood) in adults and cord blood. The group mean point (large circle) and the confidence ellipses (yellow and light blue) are shown for each cohort. The plot shows the relationship between the variables (arrows). Positively correlated variables are grouped together while negatively correlated variables are positioned on opposite sides of the plot origin. The length of the arrow displays the size of the contribution of each variable to each dimension of the plot (cos2). Dim1 = PC1 and Dim2 = PC2. Data in **(B)** and **(C)** is shown for *n* = 14 adults and *n* = 11 cord blood samples. Abbreviations: EOS: eosinophils; GRN: granulocytes; ILC: innate lymphoid cells; LYM: lymphocytes; MAIT: mucosal invariant T cells; MON: monocytes; NEU: neutrophils; NK: natural killer cells NKT: natural killer T-like cells; Tc2: type-2 CD8 + T cells; Th2: type-2 CD4 + T cells.

We first sought a holistic view of the different cell populations in cord and adult blood. Following staining for surface markers, flow cytometry data, were gated on single, live, CD45^+^ leukocytes, and were analyzed by Uniform Manifold Approximation and Projection (UMAP) [28] dimensionality reduction (Fig. 1A and Supplementary Fig. S1, for individual plots). A total of 15 000 randomly selected leukocytes from two adult and nine cord blood samples were concatenated for analysis. UMAP analysis clearly described and segregated the populations of leukocytes, validating our manual gating strategy used for later analysis [26]. UMAP analysis allowed visualization of a relatively low abundance of NKT and MAIT cells, by the small size of these clusters, in cord blood plots (Fig. 1A). The diversity of populations within the cord blood cohort can be seen in the plots of lymphocytes from individual donors (Supplementary Fig. S1).

To further analyze differences and similarities between the cohorts, unsupervised hierarchical clustering was used to compare cell numbers per ml blood in individuals and displayed as a heatmap (Fig. 1B). Adult samples segregated from cord blood samples into a single cluster (Fig. 1B). Principal component analysis (PCA) was used to examine the separation and factors driving differences in the cohorts, using abundance of different populations (cell numbers per ml blood) from each individual (Fig. 1C). This analysis segregated samples according to cohort (adult vs. cord blood), and not according to sex (see Supplementary Fig. S2). Principal component PC1 explained 60% of variance and PC2 explained 15% of variance between samples. Many variables drove the separation between samples but interestingly, the variables (cell populations) with the highest contribution in PC1 (as represented by the cos2 value) were conventional T cells, followed by ILC, particularly ILC1 (Fig. 1C). Additionally, this analysis indicated a relative homogeneity among adult samples, relative to the greater diversity evident between individuals in the cord blood group, as shown by the tighter clustering of adult samples.

### Low abundance of NKT and MAIT cells, similar abundance of NK cells, and high abundance of ILCs in cord blood

**Figure 2.**
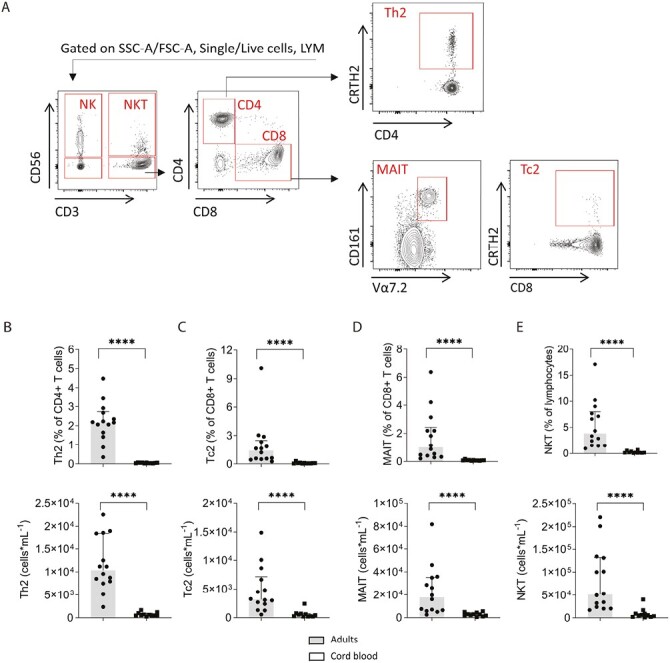
Abundance and frequencies of T and NKT-like lymphocyte subsets in blood from healthy adults and cord blood. (**A**) Representative flow cytometry for NK, NKT-like, and CD4^+^ and CD8^+^ T cells and their subsets in peripheral whole blood from a healthy adult. Lymphocytes were defined within live, single, cells using CD45 expression and SSC-A. Within the lymphocyte population, T lymphocytes (CD3^+^CD56^−^ cells) were segregated into CD4^+^ or CD8^+^ T cells. Within CD4^+^ and CD8^+^ T cells, the CRTH2 marker was used to define Th2 and Tc2 cells, respectively. Within CD8^+^ T cells, MAIT cells were defined as CD161^H^ Vα7.2^+^. Frequency and numbers of **(B)** Th2 cells among CD4 + T cells, **(C)** Tc2 cells among CD8^+^ T cells, **(D)** MAIT cells among CD8^+^ T cells, and **(E)** NKT-like cells among lymphocytes, in whole blood from healthy adults (*n* = 14) and cord blood (*n* = 11). Data shown as the median with upper interquartile range. The significance of differences between groups was determined using the Mann–Whitney test (****P < 0.0001). Abbreviations: LYM: lymphocytes; NK: natural killer cells; NKT: natural killer T-like cells; MAIT: mucosal-associated invariant T cells; Tc2: type-2 CD8 + lymphocytes; Th2: type-2 CD4 + lymphocytes.

To further understand the differences between adult and cord blood cells, particularly the lymphocytes, the major leukocyte populations were first enumerated in whole adult and cord blood following staining for surface markers and using our manual gating strategy [26]. Overall, CD45 + leukocytes were more abundant in cord blood with a median of 8.26 × 10^6^ (range 4.55 × 10^6^–1.53 × 10^7^) leukocytes per ml of cord blood compared to 4.14 × 10^6^ per ml (2.11 × 10^6^–7.29 × 10^6^) in adult blood (*P* = 0.0002). The abundance of granulocytes (including neutrophils and eosinophils), monocytes, and lymphocytes (including CD4^+^ and CD8^+^ T-cell subsets), were all significantly higher in cord blood than in adults (Supplementary Fig. S3). Monocytes and lymphocytes comprised a significantly higher proportion of live, single cells in cord blood than in adults and there was a higher proportion of eosinophils within the granulocyte population in cord blood than in adults (median values of 9.8% vs. 2.6%; Supplementary Fig. S3B and S3C). Within the T-cell population, there was no significant difference in the ratio of the CD4^+^ and CD8^+^ subpopulations (Supplementary Fig. S3D). Expression of CRTH2 was used to define type 2 T-helper cells (Th2 cells) within the CD56^−^CD3^+^CD4^+^ population, and type 2 cytotoxic T cells (Tc2 cells) within the CD56^−^CD3^+^CD8^+^ population (Fig. 2A). In contrast to the higher overall T-cell abundance in cord blood, Th2 and Tc2 subpopulations were far less abundant in cord blood than in adults (median abundance 20-fold and 10-fold lower than in adults, respectively) and they constituted a lower proportion of CD8^+^ T cells (Tc2 cells) or CD4^+^ T cells (Th2 cells), respectively, than in adults (*P* < 0.0001; Fig. 2B and C).

We next enumerated subsets of innate lymphocytes in cord and adult blood. MAIT cells are CD3^+^CD8^+^CD161^H^ lymphocytes which in humans express Vα7.2-Jα33/12/20 and Vβ2 or Vβ13 TCR chains [29]. MAIT cells were defined as CD161^H^ Vα7.2^+^ cells within the CD56^−^CD3^+^CD8^+^ T-cell population (Fig. 2A). The median abundance of MAIT cells was 7-fold lower in cord than in adult blood (Fig. 2D). Within the lymphocyte gate, NKT-like cells were identified as CD56^+^CD3^+^ cells, and the median abundance of these innate T lymphocytes was 9-fold lower in cord blood compared to adults (Fig. 2E).

**Figure 3.**
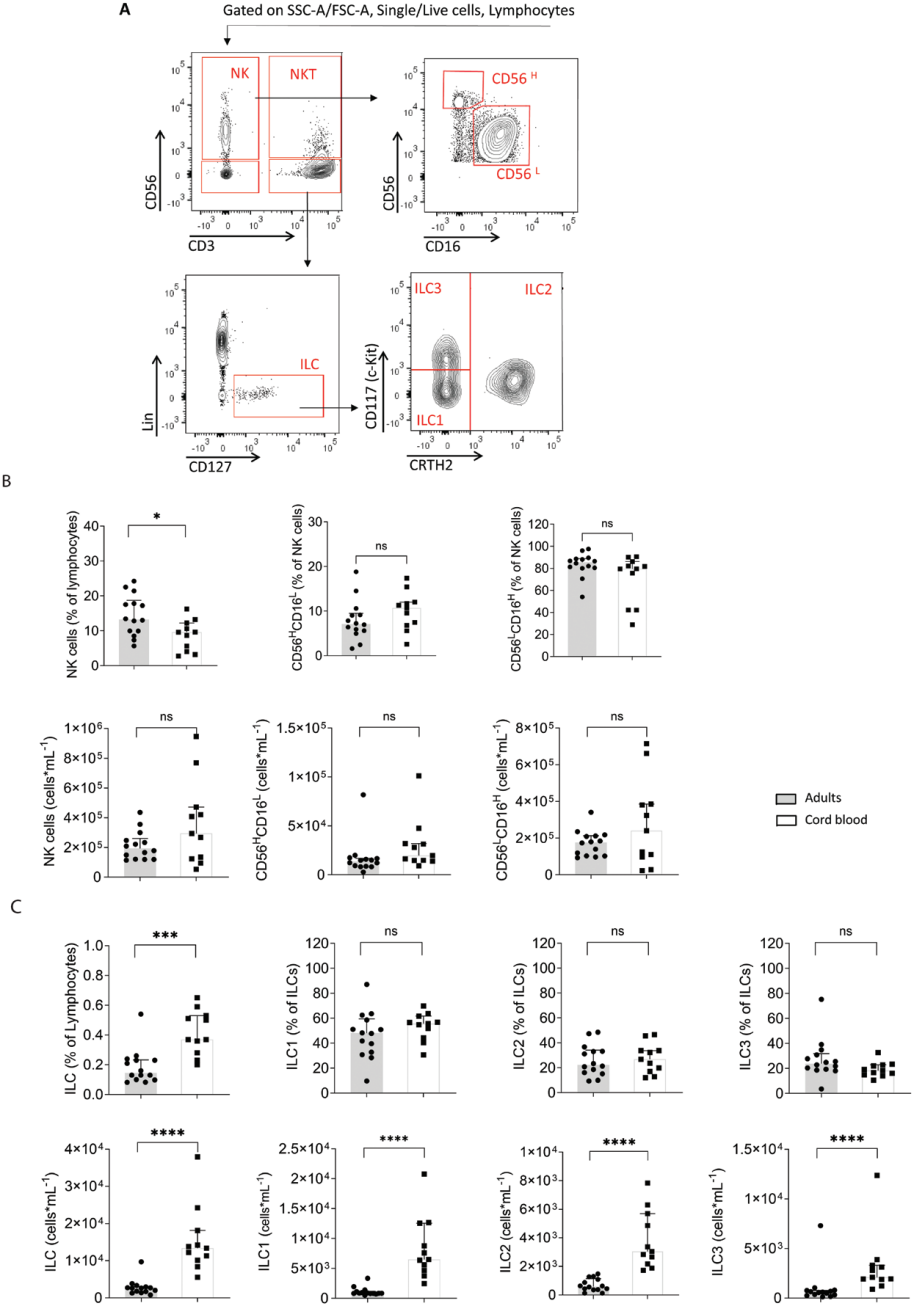
Abundance and frequencies of NK cells and ILCs in blood from healthy adults and cord blood. (**A**) Representative flow cytometry gating for NK cells and ILC in peripheral whole blood from a healthy adult. Lymphocytes were defined within live, single, cells using CD45 expression and SSC-A. NK cells were defined as CD56^+^CD3− lymphocytes. Within the NK cell population, CD56^H^CD16^L^ and CD56^L^CD16^H^ subsets were defined. NKT-like cells were gated within the lymphocyte population as CD56^+^CD3^+^ cells. ILCs were delineated within the lymphocyte gate as CD3^−^ CD56^−^ lin^−^ and CD127^+^ cells. Within the ILC population, ILC1 were defined as CD117^−^CRTH2^−^, ILC2 as CRTH2^+^ CD117^int^, and ILC3 as CD117^+^CRTH2^−^. **(B)** Frequency and numbers of NK cells among lymphocytes and frequency and numbers of the CD56^H^CD16^L^ and CD56^L^CD16^H^ subset**s** among NK cells. **(C)** Total numbers and frequency of ILCs among lymphocytes and ILC1, ILC2, and ILC3 among total ILC in whole blood from healthy adults and cord blood. Data are shown as the median with upper interquartile range. Data is shown for *n* = 14 adults and *n* = 11 cord blood samples. The significance of differences between groups was determined using the Mann–Whitney test (ns, not significant; *P < 0.05, ***P < 0.001, ****P < 0.0001). Abbreviations: Lin: lineage; NK: natural killer cells, NKT: natural killer T: like cells.

NK cells were defined as live, single, CD45^+^CD56^+^CD3^−^ lymphocytes (Fig. 3A). NK cells constituted a significantly lower proportion of the lymphocyte population in cord blood (median 9.58%) than adult peripheral blood (median 13.2%), however, in contrast to NKT and MAIT cells, the abundance of NK cells was not significantly different in cord blood (Fig. 3B). Within the NK cell population, the CD56^H^CD16^L^ and CD56^L^CD16^H^ subsets were of similar abundance. Of note, however, in some cord blood samples, some of CD45^+^CD56^+^CD3^−^ cells, had low expression of both CD16 and CD56 and fell outside of the gates used to define the NK cell subsets (Supplementary Fig. S4 for individual sample plots and FMO controls).

Next, different ILC populations were enumerated. ILCs lack antigen-specific receptors and instead are activated *via* germ-line encoded receptors or soluble mediators such as cytokines and alarmins released following tissue damage [7, 30]. Once activated they can rapidly produce cytokines and have been classified into three groups: ILC1, ILC2, and ILC3, which mirror type-1, type-2, and type-17 adaptive T cells [9, 31, 32]. ILCs were defined within live, single, CD45 + lymphocytes as CD3^−^CD56^−^lineage^−^CD127^+^ cells (Fig. 3A). Within this population, ILC1 were defined as CD117^−^CRTH2^−^, ILC2 as CRTH2^+^CD117^int^ and ILC3 as CD117^+^CRTH2^−^. The absolute number of ILCs per ml was significantly higher at birth (median 1.34 × 10^6^ cells per ml blood compared to 2.69 × 10^3^ cells per ml in adults (*P* < 0.0001)) and the proportion of the lymphocyte population that was made up of ILC was higher in cord blood (Fig. 3C). All subsets of ILC were more abundant in cord blood than in adults and the relative frequencies of the different subsets did not differ between adult and cord blood.

### Low capacity for cytokine production in NKT-like lymphocytes, but maturity of cytokine production in NK cells and ILC from cord blood compared to adults

**Figure 4.**
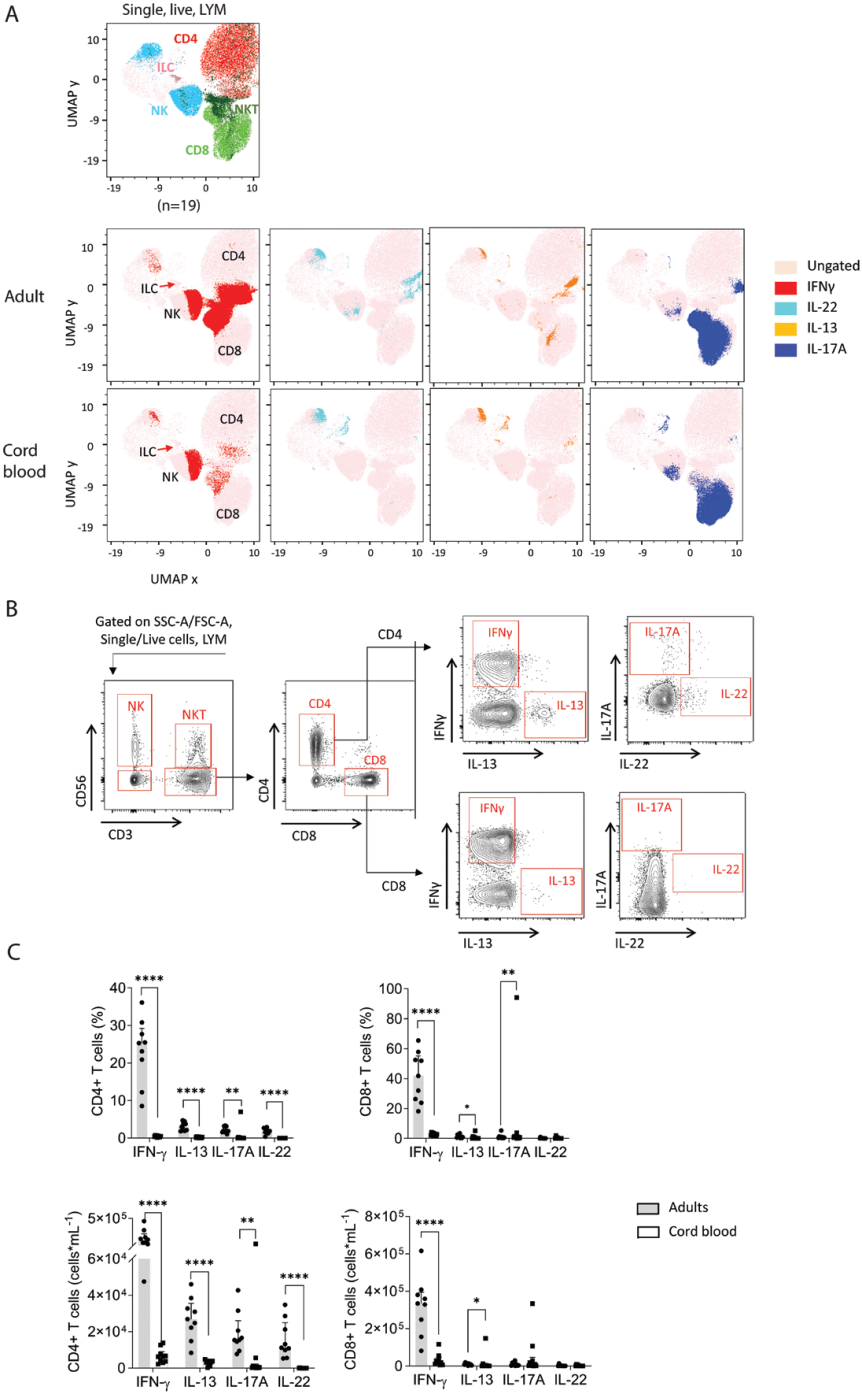
Comparison of cytokine production in lymphocytes from adult and cord blood PBMCs. Isolated PBMC from adults or cord blood were stimulated with PMA and ionomycin in the presence of GolgiPlug. Cells were analyzed for their cytokine production by intracellular staining and flow cytometry. **(A)** UMAP analysis was performed on data concatenated from 30 000 randomly selected live, single, lymphocytes from each sample. The manual gating strategy for different cell populations was used to classify global UMAP clusters into ILC (brown), NK cell (blue), NKT cell (dark green), CD4 + (red), and CD8 + (light green) T-cell populations. Cytokine production is shown on the UMAP projections indicating the production of IFN-γ (red), IL-22 (light blue), IL-13 (orange), and IL-17A (blue) within different cell clusters. **(B)** For analysis of cytokine production within T cells, lymphocytes were defined using SSC-A and FSC-A within single, live cells, and T cells defined as CD3 + CD56− lymphocytes. Illustrative flow cytometry data for IFN-γ, IL-13, IL-17A, and IL-22 production in CD4^+^ and CD8^+^ T cells from an adult donor. **(C)** Frequency and total numbers of CD4^+^ T cells and CD8^+^T cells expressing IFN-γ, IL-13, IL-17A, and IL-22. Data are shown as the median with upper interquartile range for *n* = 10 adults and *n* = 9 cord blood samples. The significance of differences between groups was determined using the Mann–Whitney test (*P < 0.05, **P < 0.01, and ****P < 0.0001). Abbreviations: LYM: lymphocytes; NK: natural killer cells; NKT: natural killer T cells.

To determine the functional capacity of different lymphocyte populations, we determined their production of different cytokines. PBMC were stimulated with PMA and ionomycin, followed by intracellular staining for the cytokines IFNγ, IL-13, IL-17A, and IL-22, the hallmarks of different T cell and ILC populations. For global analysis of cytokine production, UMAP analysis was applied to 570 000 randomly selected single, live lymphocytes (gated using SSC-A and FSC-A) concatenated from 30 000 cells from each of *n* = 9 adults and *n* = 10 cord blood samples that had been stained for intracellular cytokine production (Fig. 4A). The UMAP plots demonstrate enriched regions of IFNγ, IL-13, IL-17A, and IL-22 production in CD4^+^ and CD8^+^ T cells in adults and weaker responses in cord blood cells. In contrast, IFNγ expression within the NK cell cluster in cord blood samples was clearly visible. The apparent IL-17 production shown on the UMAP of cord blood cells was due to a high level of IL-17 expression in the lymphocytes of one outlying cord blood sample, which was included in the concatenated data.

Next, a manual gating strategy was employed to analyze cytokine production in different lymphocyte populations. Illustrative data and the full gating strategy using stimulated and unstimulated cells and FMO controls are shown in Supplementary Figs. S5–S7. First, T cells (CD3^+^CD56^−^) were segregated into CD4^+^ or CD8^+^ T cells (Fig 4B). As seen in the UMAP analysis, a much higher proportion of CD4^+^ T cells produced IFNγ, IL-13, and IL-17A in adults compared to cord blood and the number of CD4^+^ T cells producing IFNγ, IL-13, IL-17A, and IL-22 was also significantly higher in adults (Fig. 4C). Similarly, a median of 37% of CD8^+^ T cells were positive for IFNγ in adults compared to a median of only 3% in their cord blood counterparts (Fig. 4C).

**Figure 5.**
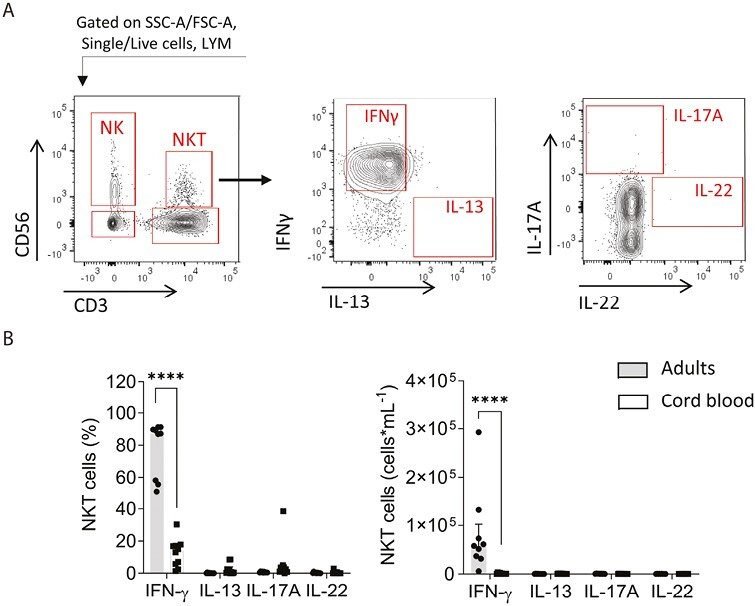
Comparison of cytokine production in NKT-like cells from adult and cord blood PBMCs. Isolated PBMC from adults or cord blood were stimulated with PMA and ionomycin in the presence of GolgiPlug. Cells were analyzed for their cytokine production by intracellular staining and flow cytometry. **(A)** For analysis of cytokine production lymphocytes were defined using SSC-A and FSC-A within single, live cells, and NKT-like cells defined as CD3 + CD56 + lymphocytes. Illustrative flow cytometry data for IFN-γ, IL-13, IL-17A, and IL-22 production in NKT-like cells from an adult donor. **(B)** Frequency and total numbers of NKT-like cells expressing IFN-γ, IL-13, IL-17A, and IL-22. Data are shown as the median with upper interquartile range for *n* = 10 adults and *n* = 9 cord blood samples. The significance of differences between groups was determined using the Mann–Whitney test (****P < 0.0001). Abbreviations: LYM: lymphocytes; NK: natural killer cells; NKT: natural killer T cells.

Next, we sought to determine the capacity for cytokine production in innate lymphocyte populations. NKT-like cells were defined within the lymphocyte gate as CD56^+^CD3^+^ cells (Fig. 5A). These lymphocytes predominantly produced IFNγ in adult blood, however, in cord blood the frequency of NKT-like cells that could produce IFNγ was much lower (Fig. 5B), which, combined with the low abundance of these cells, resulted in a 149-fold lower median abundance of NKT-like cells capable of producing IFN-γ in cord blood (Fig. 5B).

**Figure 6.**
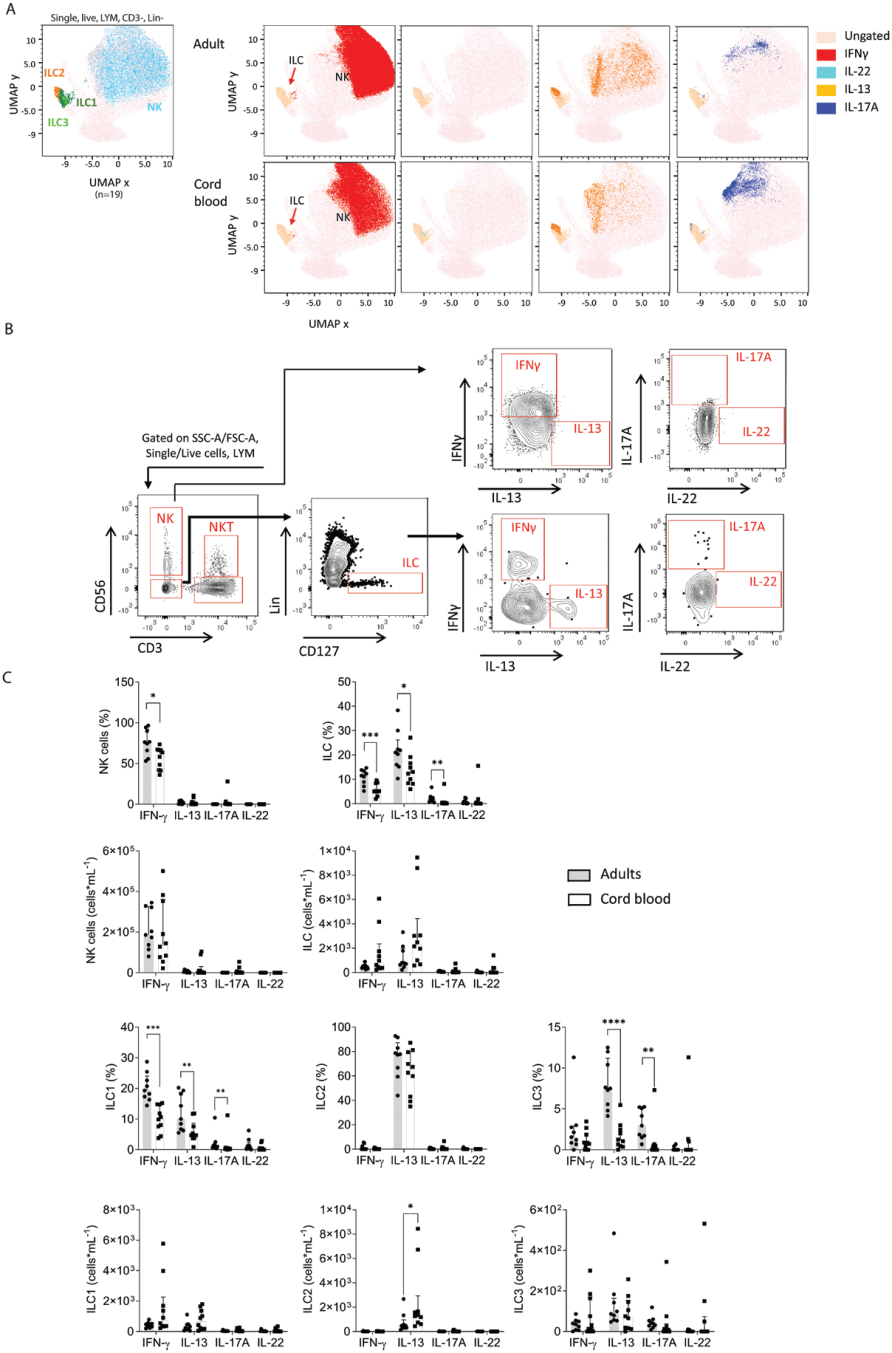
Comparison of cytokine production in stimulated NK cells and ILCs from adult and cord blood PBMC. PBMC were stimulated with PMA and ionomycin in the presence of GolgiPlug, and cell populations analyzed for their cytokine production by intracellular staining and flow cytometry. **(A)** Data were concatenated from 30 000 randomly selected single live CD3^−^ lin^−^ lymphocytes from adults (*n* = 9) and cord blood (*n* = 10) for UMAP analysis. A manual gating strategy was used to classify global UMAP clusters into NK cells (blue), ILC1 (green), ILC2 (orange), ILC3 (light green). UMAP projections indicate the production of IFN-γ (red), IL-22 (light blue), IL-13 (orange), and IL-17A (blue) in cells from adults and cord blood. **(B)** Representative flow cytometry data for IFN-γ, IL-13, IL-17A, and IL-22 expression in NK cells and ILC in PBMC from an adult donor. Lymphocytes were defined within live, single cells using FSC-A and SSC-A. Within the lymphocyte gate, NK cells were defined as CD56^+^CD3− cells and ILCs as CD3^−^ CD56^−^ lin^−^ CD127^+^ cells, and the populations of IFN-γ, IL-13, IL-17A, and IL-22 positive cells were defined using a biaxial gating strategy. **(C)** Frequency and total numbers per ml of NK cells, ILCs, ILC1, ILC2, and ILC3 expressing IFN-γ, IL-13, IL-17A, and IL-22. Data are shown as the median with upper interquartile range. The significance of differences between groups was determined using the Mann–Whitney test (*P < 0.05, **P < 0.01, ***P < 0.001, and ****P < 0.0001)

For a detailed analysis of cytokine production within innate lymphocyte populations, UMAP analysis was performed on single, live, CD3^−^lin^−^ lymphocytes using 30 000 cells per subject from *n* = 9 adult and *n* = 10 cord blood samples. This analysis allowed visualization of pronounced IFNγ production by NK cells, robust IL-13 production in ILC2 from both adults and cord blood, and enrichment of a region corresponding to IL-22 production in ILC3 in cord blood (Fig. 6A).

Further analysis of cytokine production within NK cells using manual gating (Fig. 6B and Supplementary Figs. S5 and S6) revealed that frequencies of IFNγ^+^ cells were slightly lower in cord blood than in adults (median 58% positive vs. 76%; Fig. 6C). However, in striking contrast to T lymphocytes, when cell numbers were considered, no significant differences were observed in the total numbers of cytokine secreting NK cells per ml blood in adults and cord blood (Fig. 6C).

Cord blood ILC were capable of producing their hallmark cytokines following *ex vivo* stimulation, however, there were some differences to those from adult blood (Fig. 6B and 6C). The frequency of IFNγ production was lower within ILCs in cord blood than in adults. The frequencies of IL-13 and IL-17-producing ILCs were also slightly lower, however, when numbers were taken into account the abundance of cytokine-secreting ILCs per ml was not significantly different between adults and cord blood (Fig. 6C).

When different subsets were considered, ILC1 demonstrated a slightly lower frequency of IFNγ production in cord blood compared to adults, but the abundance of IFNγ producing ILC1 was not significantly different (Fig. 6C). The frequency of IL-13 production in ILC2 was comparable to adults, demonstrating that these innate lymphocytes are functionally fully mature, in terms of cytokine production, in cord blood. Higher numbers of ILC2 in cord blood meant that IL-13-producing ILC2 were more abundant (Fig. 6C). The frequency of IL-13 and IL-17-producing cells among ILC3 was higher in adults, and due to higher numbers of ILC in cord blood, abundance of IFNγ, IL-13 and IL-17A and IL-22 producing ILC3 was not significantly different in adults and cord blood (Fig. 6C).

### Maturity of granzyme B production in NK cells but not NKT-like or T-cells in cord blood

**Figure 7.**
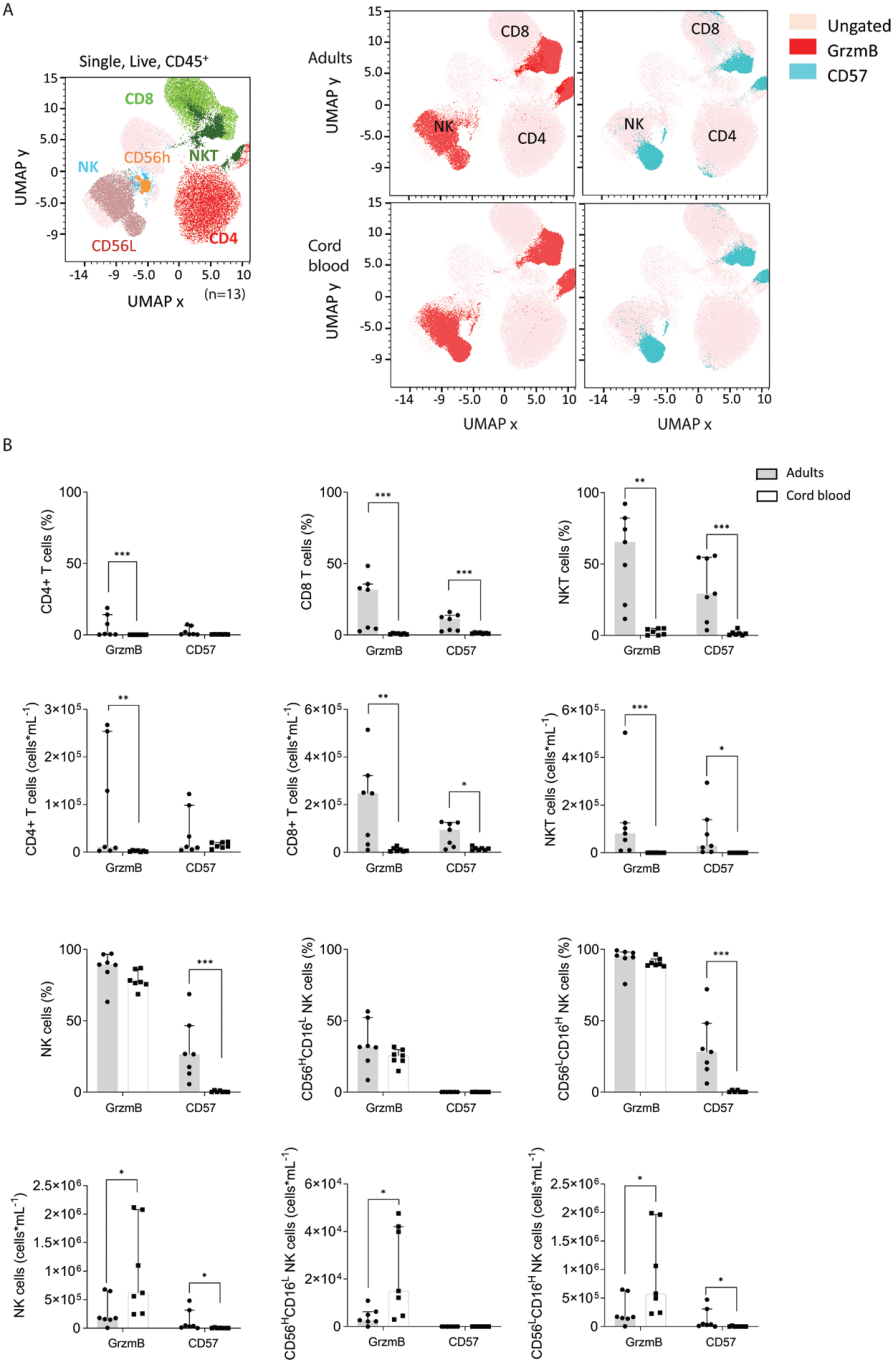
Comparison of Granzyme B and CD57 expression in lymphocytes from healthy adult and cord blood PBMC. (**A**) 25,000 randomly selected CD45^+^ lymphocytes from each subject were concatenated for UMAP analysis and the manual gating strategy used to identify clusters corresponding to CD4^+^ T cells (red), NKT -like cells (dark green), CD8^+^ T cells (light green), NK cells (blue), CD56^H^ NK cells (orange), and CD56^L^ NK cells (light brown). UMAP projections indicating the production of GrzmB (red) and CD57 (blue) expression in adult and cord blood lymphocytes. **(B)** Frequency and total numbers of CD4^+^ and CD8^+^ T cells and NKT-like cells expressing granzyme B and CD57 in PBMC from healthy adults and cord blood. **(C)** Frequency and total numbers of NK cells, CD56^H^CD16^L^ NK cells, and CD56^L^CD16^H^ NK cells expressing granzyme B and CD57 in PBMC from healthy adults and cord blood. Data are shown as the median with upper interquartile range. Significance of differences between groups was determined by Mann–Whitney test (*P < 0.05, ***P < 0.001). Abbreviations: GrzmB: granzyme B; ILC—innate lymphoid cells; NK: natural killer cells; NKT: natural killer T-like cells; UMAP: uniform manifold approximation and projection.

Lymphocytes were further characterized by flow cytometry in adult and cord blood to determine their cytotoxic capacity (granzyme B production) and expression of CD57, a marker of terminal differentiation and maturation. Striking differences in granzyme B and CD57 expression in lymphocytes in adults and cord blood were demonstrated by UMAP analysis on 325 000 lymphocytes (defined on SSC-A and CD45 expression) concatenated from 25 000 randomly selected cells from each of seven subjects from each cohort (Fig. 7A). Notably, although granzyme B expression was prominent in NK cells from adults and cord blood, within CD4^+^ and CD8^+^ lymphocytes, CD57 and granzyme B are clearly seen within adult but not cord blood T cells.

A manual gating strategy was then used to further analyze granzyme B and CD57 expression in cord blood and adult cells (Supplementary Figs. S8 and S9). As suggested by the UMAP analysis, the frequency of CD4^+^ and CD8^+^ T cells that expressed granzyme B and CD57 was much higher in adults than cord blood, with the exception of CD57 expression on CD4^+^ T cells (Fig. 7B). This resulted in higher numbers of granzyme B positive CD4^+^ and CD8^+^ T cells in adults than cord blood (5-fold and 41-fold higher median numbers per ml, respectively) and higher numbers of CD57^+^CD8^+^ T cells (6-fold higher median numbers per ml). Analysis of NKT-like cells also revealed significantly lower expression of granzyme B and CD57 in this innate lymphocyte population, with 785-fold higher median numbers of granzyme B^+^ NKT-like cells and 357-fold higher median numbers of CD57^+^ NKT-like cells per ml blood in adults (Fig. 7B).

In contrast, the frequency of NK cells that expressed granzyme B was similar in adults and cord blood, but the slightly higher numbers of NK cells in cord blood resulted in a significantly higher abundance of granzyme B positive NK cells in cord blood (Fig. 7C and Supplementary Fig. S9). NK cells were segregated into CD56^H^CD16^L^ and CD56^L^CD16^H^ subsets and in both adults and cord blood granzyme B was predominantly expressed in the CD56^L^ population. CD57 expression was predominantly in the CD56^L^CD16^H^ subpopulation and was much higher in adults, suggesting that circulating NK cells are not fully terminally differentiated in cord blood (Fig. 7C and Supplementary Fig. S9).

### Discussion

In this study, we set out to determine the differences in abundance and functional maturity of innate lymphocytes at birth compared to adulthood. Our work revealed clear differences in different innate lymphocyte subsets; those present in low abundance (NKT cells and MAIT cells) with low cytokine production following stimulation (NKT cells) and those with similar abundance and capacity for cytokine and granzyme B production in early life to that of adults (NK cells and ILCs). These differences in innate lymphocyte function occur in the context of circulating adaptive T lymphocytes that are present at high abundance in cord blood but which produce low levels of cytokines when stimulated. Furthermore, a greater heterogeneity in cell abundance between individual donors was demonstrated in cord blood than in adults.

We find far lower numbers of invariant NKT-like and MAIT cells in cord blood. The difference in abundance of MAIT cells has been reported by others to be 30 times higher in peripheral blood of adults than in newborns [29], expanding around 10-fold during the first year of life [33]. Here we additionally demonstrate the striking differences in the production of IFNγ by NKT-like cells in cord blood. Acquisition of the microbiota is a key event in neonates for immune maturation after birth and NKT and MAIT cell development is thought to be influenced by commensal organisms and driven by microbial metabolites following colonization with the microbiota [13, 19, 34]. Colonization may also drive the expansion of innate γδ T cells [35], the development of the B-cell repertoire [36], and the frequency of intestinal group 3 innate lymphocytes [37]. One limitation of this study is that we did not include a marker for γδ T cells. Others have found these lymphocytes to be present at a low abundance in early life and to exhibit a different phenotype and TCR repertoire to those found in adults [38, 39]. As γδ T cells differ in pre-term infants, further characterization of their phenotype and function in early life is an important area for further study [40].

The abundance of NK cells was less influenced by age than that of MAIT cells or NKT cells, and we found a similar proportion of the CD56^H^CD16^L^ and CD56^L^CD16^H^ subsets in early life as in adults. NK cells produced IFNγ at only slightly lower levels in cord blood, and the proportion of NK cells that expressed granzyme B was not different from that of adults. We also report that cord blood NK cells had very low expression of CD57, which defines terminally differentiated mature NK cells [41, 42]. Others have similarly reported differences in phenotype and function of cord blood NK cells, suggesting that they are not fully functionally mature, although not all studies agree on how they differ from adult NK cells [43]. In murine NK cells, an impaired anti-viral activity *in vivo* in early life was found to be regulated by TGF-β [44]. NK cells have been found to be the predominant innate lymphocyte population in the infant gut where they express higher amounts of perforin and granzyme than those from adults [45]. Furthermore, the selective placental transfer of antibodies that activate NK cells supports the concept that NK cells are functionally important in early-life protection against infection [46].

ILC, although most abundant at mucosal surfaces, can be detected in peripheral blood. We found increased numbers of all ILC subsets in cord blood, in accordance with others [18]. The ratio of the different ILC subsets did not differ from that of adults. The functional differences in ILC in cord blood have not previously been well explored. We found that ILC1 and ILC2 from cord blood could produce their hallmark cytokines IFNγ and IL-13, respectively, and although the proportion was lower than in adults, due to their higher abundance, the total number of cytokine-producing ILC per ml of cord blood was comparable to that of adults. Others mapped the abundance of ILCs in peripheral blood from the age of 5 to 74 years and report a general decline with age in all ILC populations [47]. Early life is a period of immunological development and of susceptibility to infection [3] and ILC plays many roles in promoting and regulating immune responses, particularly at mucosal sites [7, 8]. Murine studies have defined important roles for ILC in the postnatal period. In mice, ILC2 are abundant in the lung during the post-natal phase of development and ILC2 numbers and cytokine production are enhanced following respiratory viral infection in mice [21, 48–51]. In mice, there is also increasing evidence for the importance of ILC3 in early-life immunity [52]. These cells expand in offspring in response to signals from the maternal microbiota [37]. In a murine model of neonatal gut inflammation, ILC3 secrete cytokines and promotes pathology in the intestine [24]. The gut microbiota was found to promote trafficking of ILC3 to the lung in neonatal mice where they develop under the influence of the local microenvironment and protect against bacterial pneumonia [53, 54]. In the context of the known differences in the adaptive immune system in early life, innate immunity including ILC, may play a central role in early-life immune responses, particularly at barrier sites, and influence immunity later in life [2, 3].

The differences we report in innate lymphocyte abundance and function in early life occur in the context of differences in adaptive immunity. Conventional CD3^+^ T-cell abundance was found to be higher in cord blood, with a similar ratio of CD4^+^ and CD8^+^ cells to that of adults. We observed significantly lower production of all cytokines studied in both the CD4^+^ and CD8^+^ compartments in cord blood and the proportion of T cells that expressed granzyme B and the maturation marker CD57 was also far lower in cord blood. CD4^+^ T helper cell responses in early life can be biased towards Th2 memory formation in murine models and limited type I responses in human neonatal T cells have been reported [3, 55, 56]. Based on CRTH2 expression and low levels of detection of all cytokines studied, we did not see a type 2 cytokine bias in cord blood T cells following the polyclonal stimulation used. The low production of cytokines in cord blood T cells likely reflects an abundance of recent thymic emigrants and naïve T cells and our data is consistent with others who report that low type 2 cytokine and CXCL8 production is a characteristic of CD4^+^ T cells in early life [57]. Furthermore, our data are from polyclonal stimulation of whole blood and therefore do not reflect the capacity of the adaptive immune responses in infants to develop diverse T-cell responses under different circumstances [3]. The numbers of type-2 CD8^+^ cells were also low in cord blood. In adults, these cells have been associated with severe eosinophilic asthma [58], but the factors regulating the development of these cells and association with allergic disease in early life are unclear.

Much of our knowledge of neonatal immunity comes from studies of neonatal mice, which may not fully represent the maturational trajectory of human infants. A strength of this study is in the use of techniques optimized for small volumes of blood available from human samples. A limitation of our study is that it uses cord blood rather than peripheral blood from older infants and babies and considerable changes to the frequencies of leukocyte populations and plasma proteins following birth and during early life have been reported [59]. Nevertheless, cord blood can offer important insights into differences in immune cells in early life. A further limitation of this study is the use of PMA and ionomycin to stimulate the blood samples. While this demonstrates the potential of different populations to produce cytokines, cells may become overstimulated and further study of responses of different lymphocyte populations to their physiological stimuli is warranted.

Together our data suggest that in early life NK cells and ILCs are relatively mature in terms of their capacity to act as an abundant source of cytokine production, in the context of relatively immature and inexperienced T cell and NKT-cell populations. Further understanding of pre- and post-natal factors that influence the maturation of innate lymphocytes will be important for our understanding of early life immunity, offer new insights into the pathogenesis of early life disease, help to identify biomarkers that could predict the risk of disease, and support the development of new therapeutics.

## Supplementary Material

uxad094_suppl_Supplementary_Data

## Data Availability

The data underlying this article will be shared on reasonable request to the corresponding author.

## Abbreviations:

DMSO: dimethylsulphoxide
FCS: fetal calf serum
FMO: fluorescence minus one
FSC: forward scatter
IFN: interferon
ILC: innate lymphoid cell
IL: interleukin
MAIT cells: mucosal-associated invariant T cell
near-IR: near-infrared
NK cell: natural killer cell
NKT cell: natural killer T cell
PBMC: peripheral blood mononuclear cells
PCA: principle component analysis
PMA: phorbol 12-myristate 13-acetate
SSC: side scatter Tc2 cells: type 2 cytotoxic T cells
TCR: T-cell receptor
Th2 cells: type 2 T helper cells
UMAP: Uniform Manifold Approximation and Projection.
