# Supplementary material for: Natural killer cells and innate lymphoid cells but not NKT cells are mature in their cytokine production at birth
Source: Clin Exp Immunol. 2023 Aug 9;215(1):1–14. doi: 10.1093/cei/uxad094 (PMC10776247; doi:10.1093/cei/uxad094)
Supplement: uxad094_suppl_Supplementary_Data [file uxad094_suppl_supplementary_data.pdf]

Supplementary Material

	Analysis (flow cytometry panel)		
	**Enumeration**	**Cytokine ICS**	**Grzm B / CD57**
**Cord blood cohort**			
	**n=11**	**n=10**	**n=7**
**Sex, n (%)**			
Females	3 (27)	3 (43)	2 (29)
Males	8 (73)	7 (57)	5 (71)
**Weight (kg), median (range)**	3.54 (2.64-4.20)	3.475 (2.64-4.38)	3.540 (2.64-3.93)
**Gestation at delivery (weeks), median (range)**	39.29 (38.43-39.71)	39.14 (38.43-40.14)	39.14 (38.43-39.71)
**Adults**			
	**n=14**	**n=9**	**n=7**
**Age (years), median (range)**	32.5 (23-63)	29 (23-63)	42.0 (26-63)
**Sex, n (%)**			
Females	6 (43)	5 (56)	3 (43)
Males	8 (57)	4 (44)	4 (57)

Table S1. Characteristics of the subjects used for flow cytometry staining and analysis. The range is shown as the highest and lowest value. Abbreviations used: ICS – intracellular staining; GrzmB, granzyme B.

Antibody	Clone	Supplier	Dilution used
Brilliant Violet 421™ anti-human CD127 (IL-7Rα)	A019D5	BioLegend	1:60
Brilliant Violet 510™ anti-human CD14*	63D3	BioLegend	1:60
Brilliant Violet 510™ anti-human CD19*	HIB19	BioLegend	1:120
Brilliant Violet 510™ anti-human FcεRIα*	AER-37	BioLegend	1:120
Brilliant Violet 510™ anti-human CD123*	6H6	BioLegend	1:300
Brilliant Violet 605™ anti-human CD4	RPA-T4	BioLegend	1:300
Brilliant Violet 650™ anti-human CD16	3G8	BioLegend	1:300
Brilliant Violet 711™ anti-human CD8	SK1	BioLegend	1:300
Brilliant Violet 785™ anti-human TCR Vα7.2	3C10	BioLegend	1:120
FITC anti-human CD45	HI30	BioLegend	1:120
PerCP/Cy5.5 anti-human CD117 (c-kit)	A3C6E2	BioLegend	1:40
PE anti-human CD3	OKT3	BioLegend	1:120
PE/Dazzle™ 594 anti-human CD161	HP-3G10	BioLegend	1:60
PE/Cy7 anti-human CD56 (NCAM)	5.1H11	BioLegend	1:120
Alexa Fluor® 647 anti-human CD294 (CRTH2)	BM16	BioLegend	1:40
Alexa Fluor® 700 anti-human CD66b	G10F5	BioLegend	1:300

Table S2: **Flow cytometry panel for enumeration of cell populations in whole blood**. * Lineage cocktail includes the following antibodies: CD14, CD19, CD123, FcεR1α

Antibody	Clone	Supplier	Dilution used
Brilliant Violet 421™ anti-human CD127 (IL-7Rα)	A019D5	BioLegend	1:60
Brilliant Violet 510™ anti-human CD14*	63D3	BioLegend	1:60
Brilliant Violet 510™ anti-human CD19*	HIB19	BioLegend	1:120
Brilliant Violet 510™ anti-human FcεRIα*	AER-37	BioLegend	1:120
Brilliant Violet 510™ anti-human CD123*	6H6	BioLegend	1:300
Brilliant Violet 605™ anti-human CD4	RPA-T4	BioLegend	1:300
Brilliant Violet 650™ anti-human CD56 (NCAM)	5.1 H11	BioLegend	1:40
Brilliant Violet 711™ anti-human CD8	SK1	BioLegend	1:300
PerCP/Cy5.5 anti-human CD117 (c-kit)	A3C6E2	BioLegend	1:40
PE anti-human CD3	OKT3	BioLegend	1:120
Alexa Fluor® 647 anti-human CD294 (CRTH2)	BM16	BioLegend	1:40
BUV395 Mouse Anti-Human IFN-γ	B27	BD	1:50
FITC anti-human IL-22	2G12A41	BioLegend	1:50
PE/Cy7 anti-human IL-13	JES10-5A2	BioLegend	1:50
Alexa Fluor® 700 anti-human IL-17A	BL168	BioLegend	1:50

Table S3. Flow cytometry panel for intracellular cytokine staining. * Lineage cocktail includes the following antibodies: CD14, CD19, CD123, FcεR1α

Antibody	Clone	Supplier	Dilution used
Brilliant Violet 605™ anti-human CD4	RPA-T4	BioLegend	1:300
Brilliant Violet 650™ anti-human CD16	3G8	BioLegend	1:300
Brilliant Violet 711™ anti-human CD8	SK1	BioLegend	1:300
FITC anti-human CD45	HI30	BioLegend	1:120
PerCP/Cy5.5 anti-human CD57	HNK-1	BioLegend	1:50
PE anti-human CD3	OKT3	BioLegend	1:120
PE/Cy7 anti-human CD56 (NCAM)	5.1H11	BioLegend	1:120
Alexa Fluor® 700 anti-human/mouse Granzyme B	QA16A02	BioLegend	1:50

Table S4. Flow cytometry panel for Granzyme B/CD57 staining

Figure S1

A

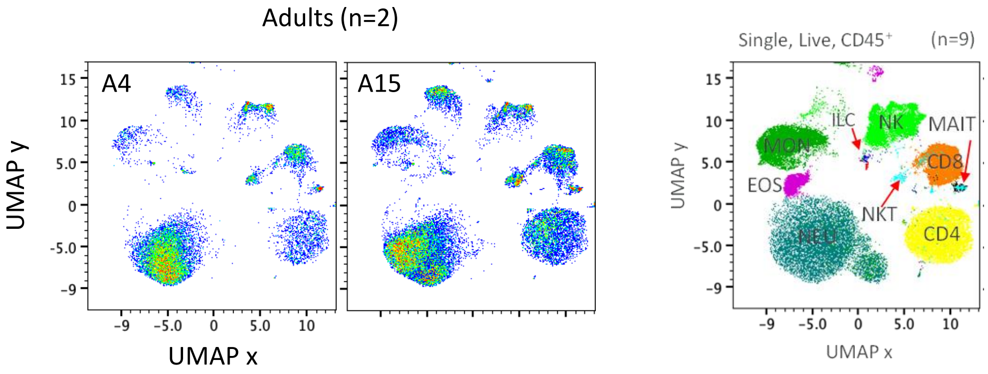

B

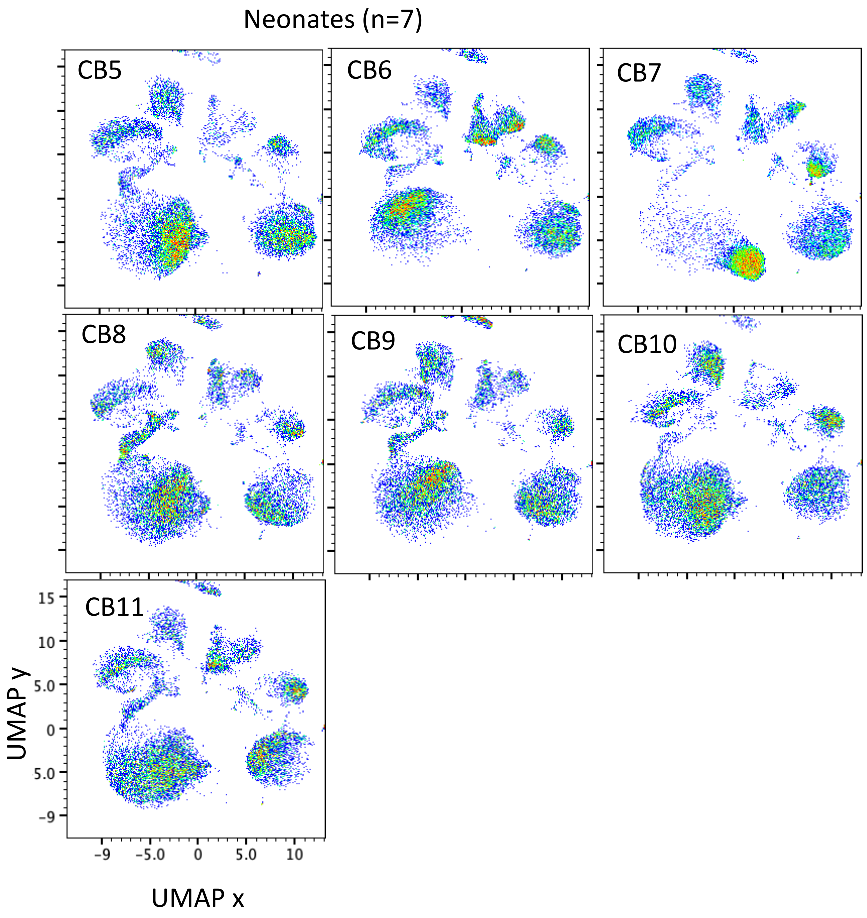

Figure S1 UMAP visualization of live CD45$^+$ cell clusters

Data analysis of individual adult **(A)** and cord blood **(B)** CD45+ leukocytes.

Figure S2

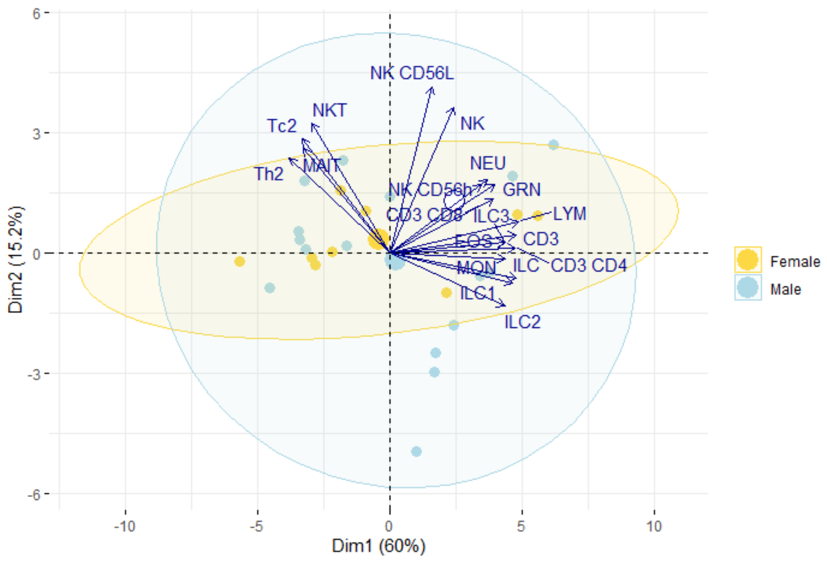

Figure S2. Adult and cord blood samples do not segregate by sex using principal component analysis of abundance of blood cell populations

Principal Component Analysis (PCA) of cell population abundance, where each individual is plotted as a small point and individuals that are similar are grouped together, showing data from adult (*n*=14) and cord blood samples (*n*=11). The sex of each individual is shown by colour. The group mean point (large circle) and the confidence ellipses (yellow and blue) are shown for each sex. The plots show the relationship between the variables (cell populations) as arrows. Positively correlated variables are grouped together while negatively correlated variables are positioned on opposite sides of the plot origin. The length of the arrow displays the size of the contribution of each variable to each dimension of the plot (cos2). Abbreviations used: NEU – neutrophils, EOS – eosinophils, MON – monocytes, LYM – Lymphocytes, NK – natural killer cells, NKT – natural killer T -like cells, MAIT – mucosal invariant T cells, ILC – innate lymphoid cells.

Figure S3

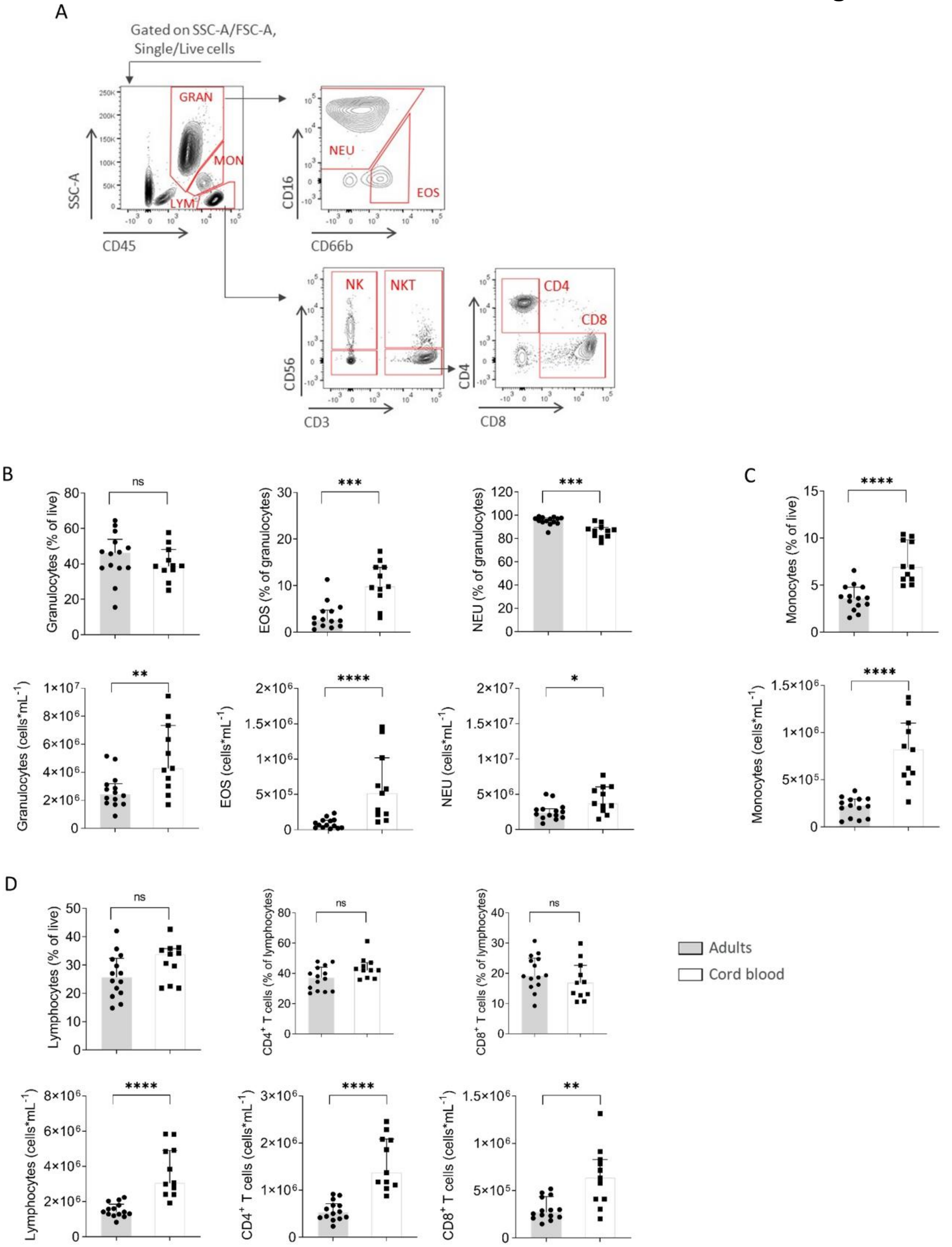
A
Gated on SSC-A/FSC-A,
Single/Live cells
GRAN
MON
LYM
SSC-A
CD45
NEU
EOS
CD16
CD66b
NK
NKT
CD56
CD3
CD4
CD8
CD4
CD8
B
C
D
Granulocytes (% of live)
EOS (% of granulocytes)
NEU (% of granulocytes)
Monocytes (% of live)
Granulocytes (cells*mL⁻¹)
EOS (cells*mL⁻¹)
NEU (cells*mL⁻¹)
Monocytes (cells*mL⁻¹)
Lymphocytes (% of live)
CD4⁺ T cells (% of lymphocytes)
CD8⁺ T cells (% of lymphocytes)
Lymphocytes (cells*mL⁻¹)
CD4⁺ T cells (cells*mL⁻¹)
CD8⁺ T cells (cells*mL⁻¹)
ns
***
****
**
*
Adults
Cord blood

Figure S3

Abundance and frequencies of granulocytes, monocytes and lymphocytes in whole blood from healthy adult and cord blood cord blood.

(A) Representative gating for major leukocyte subsets in whole blood. Granulocytes, monocytes and lymphocytes were defined within live, single cells using CD45 expression and SSC-A, and counting beads were used to obtain absolute cell counts. Within the granulocytes, neutrophils were defined as $CD16^{+}CD66b^{+/-}$ and eosinophils as $CD16^{-}CD66b^{+}$. Within the lymphocyte gate, T cells ($CD3^{+}CD56^{-}$) were segregated into $CD4^{+}$ or $CD8^{+}$ T cells. **(B)** Quantification and frequency of total granulocytes among live cells, and quantification and frequency of eosinophils and neutrophils among granulocytes**.** **(C)** Quantification and frequency of monocytes among live cells. **(D)** Quantification and frequency of lymphocytes among live cells and quantification and frequency of CD4+ and CD8+ T lymphocytes among lymphocytes. Data are from n=14 adults and n=11 cord blood samples, shown as the median with upper interquartile range. Differences between groups was determined using the Mann-Whitney test (ns, not significant; * $p < 0.05$, ** $p < 0.01$, ***$p < 0.001$ and **** $p < 0.0001$). Abbreviations used: GRAN – granulocytes, MON –monocytes, LYM – Lymphocytes, EOS – eosinophils, NEU – neutrophils.

Figure S4

Adult blood samples

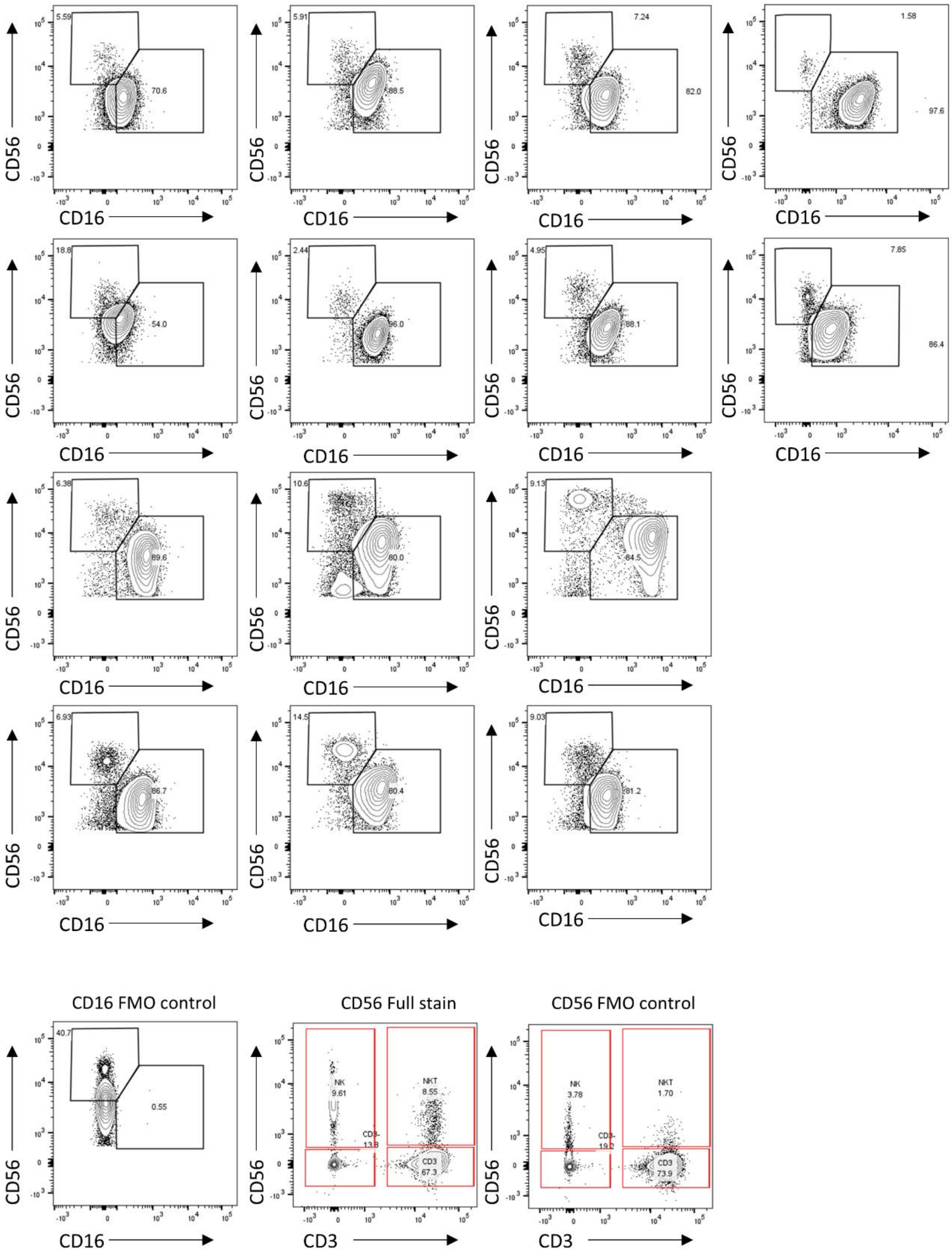

Figure S4

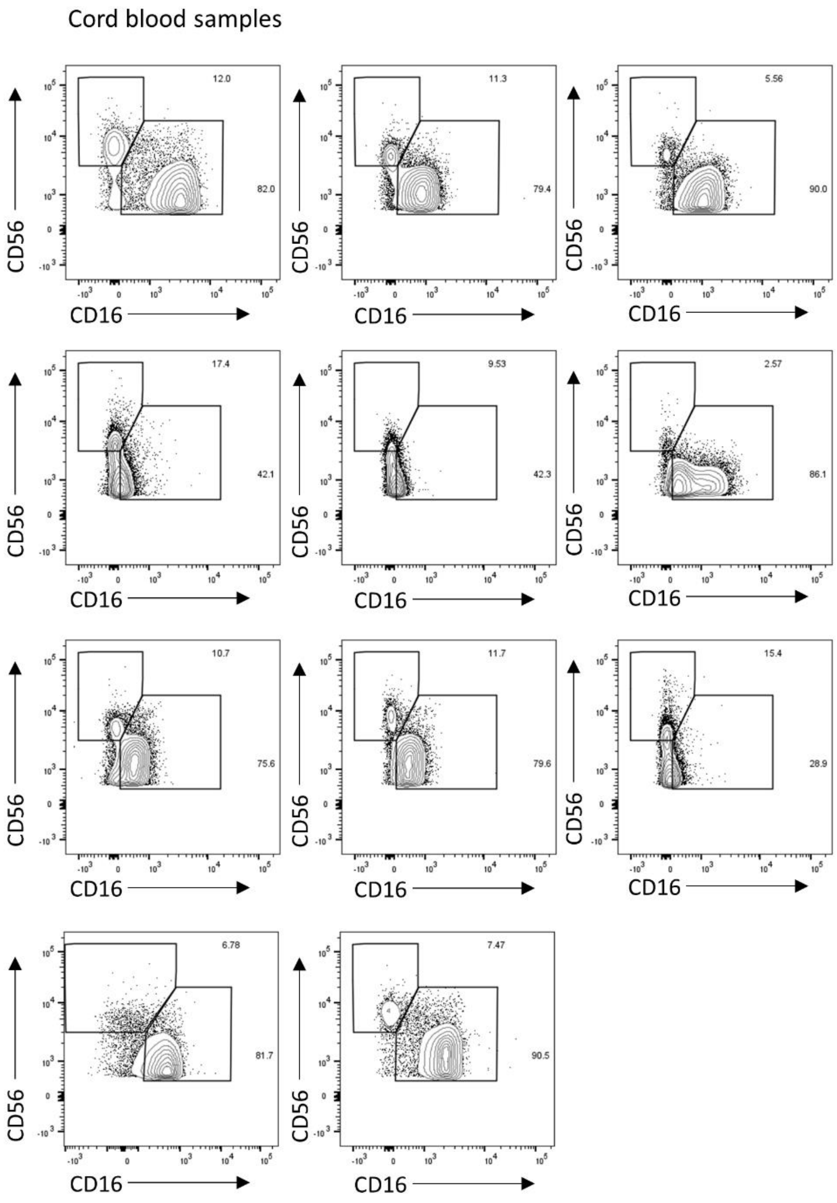

Figure S4. Expression of CD56 and CD16 on adult and cord blood NK cells. Cells were stained in whole blood and NK cells defined within the live, single, lymphocyte gate as CD3-CD56+ cells. Plots showing CD56 and CD16 staining for individual adult and cord blood donors are shown. Adult samples include examples of FMO controls for CD16 and CD56. Numbers on each plot indicate the percent of NK cells in each gate.

Figure S5

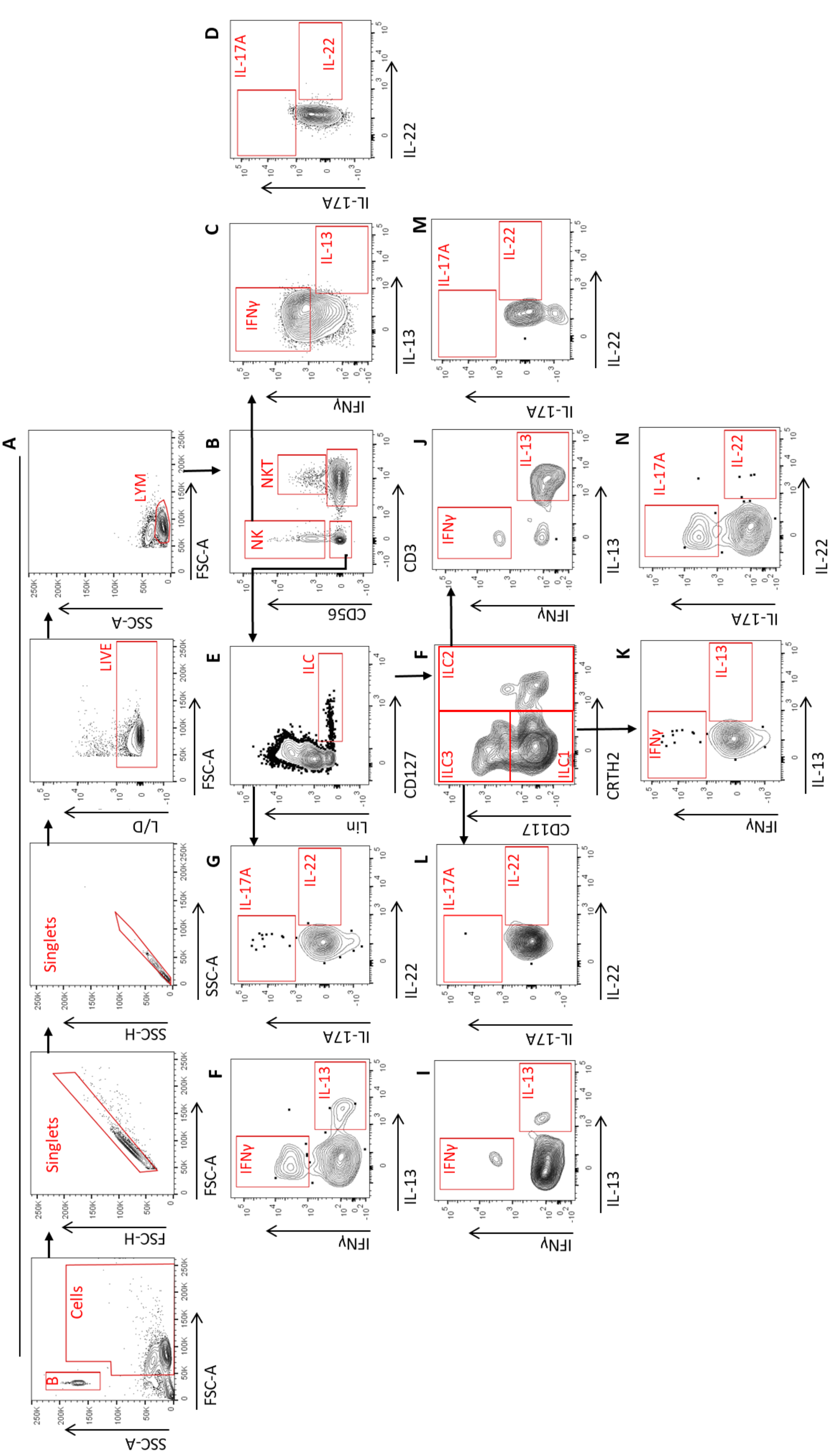
A
B
C
D
E
F
G
I
J
K
L
M
N
Cells
Singlets
LIVE
LYM
NK
NKT
ILC
ILC1
ILC2
ILC3
IFNγ
IL-13
IL-17A
IL-22
SSC-A
FSC-A
FSC-H
SSC-H
L/D
CD56
CD3
Lin
CD127
CD117
CRTH2

Figure S5. Gating strategy for identification of IFN-γ, IL-13, IL-17A and IL-22 production in ILCs, NKT -like and NK cells shown using data from stimulated adult PBMC. **(A)** Lymphocytes were gated within single, live cells using FSC-A and SSC-A. **(B)** Within the lymphocyte gate, NK and NKT-like cells were defined as $CD56^{+}CD3^{-}$ and $CD56^{+}CD3^{+}$ cells, respectively. NK cell cytokine expression was used to define **(C)** IFN-γ^{+} IL-13^{-} and IFN-γ^{-} IL-13^{+} NK cells, and **(D)** IL-$17A^{+}$IL-22^{-} and IL-$17A^{-}$IL-22^{+} NK cells. **(E)** ILCs were delineated within the lymphocyte gate as $CD3^{-}$, $CD56^{-}$, lin^{-} (CD14, CD19, CD123 and FcεRIα) and $CD127^{+}$ cells. ILCs were segregated into **(F)** IFN-γ^{+} IL-13^{-} ILCs, IFN-γ^{-} IL-13^{+} ILCs, and **(G)** IL-$17A^{+}$IL-22^{-} ILCs and IL-$17A^{-}$IL-22^{+} ILCs. **(F)** ILC1 cells were defined as $CD117^{-}CRTH2^{-}$, ILC2 cells as $CRTH2^{+}$ $CD117^{int}$ and ILC3 as $CD117^{+}CRTH2^{-}$. ILC1, ILC2 and ILC3 were further subdivided into **(I, J, K)** IFN-γ^{+} IL-13^{-}, IFN-γ^{-} IL-13^{+} or **(L, M, N)** IL-$17A^{+}$IL-22^{-} and IL-$17A^{-}$IL-22^{+} cells. Gates were defined by comparing stimulated PBMC to an unstimulated control (Figure S5) and fluorescence minus one (FMO) staining controls (Figure S6). Abbreviations used: NK – natural killer cells, NKT – natural killer T -like cells, ILC – innate lymphoid cells, B – counting beads, LD – live/dead, LYM – lymphocytes.

Figure S6

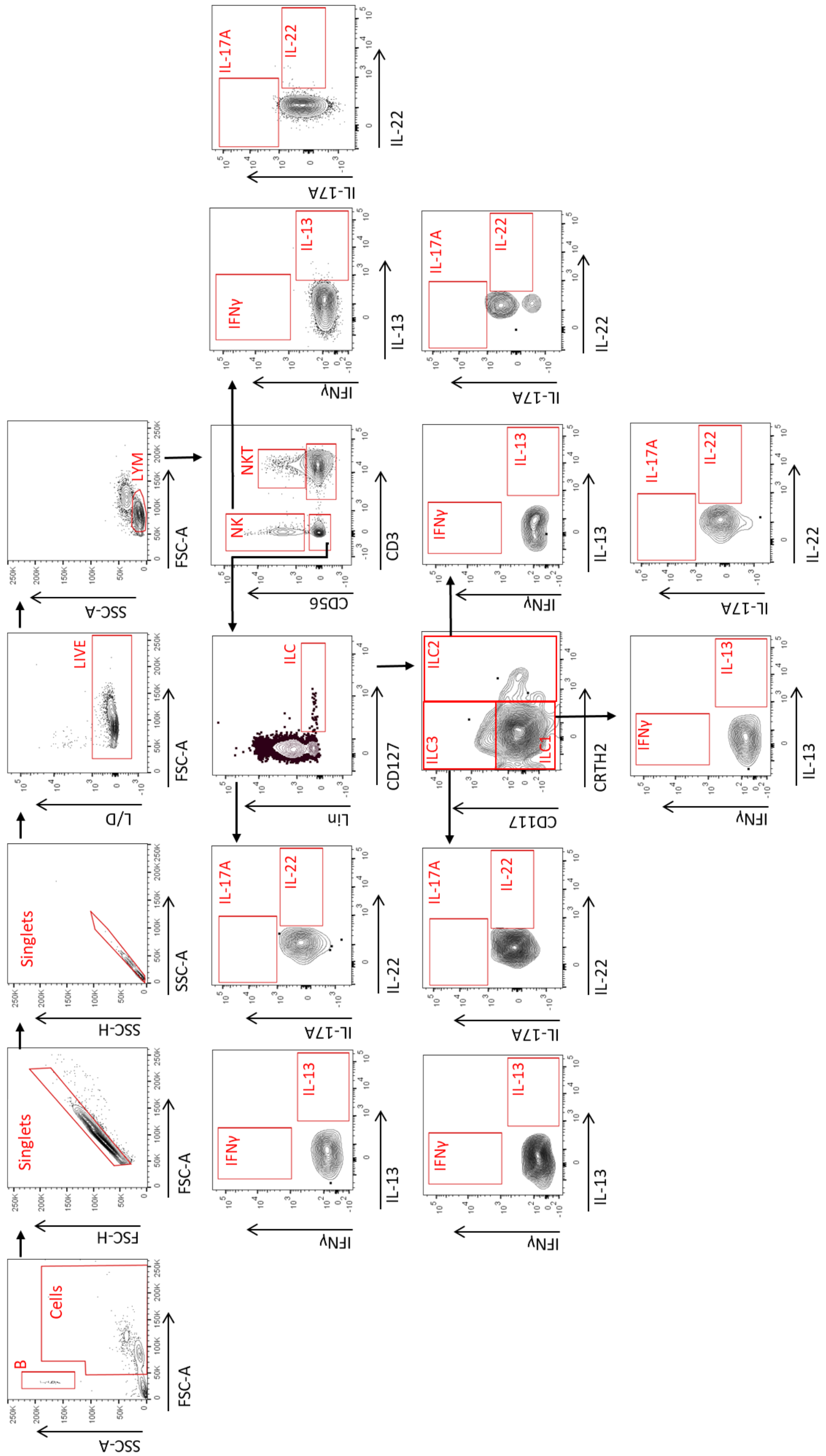
B
Cells
SSC-A
FSC-A
Singlets
FSC-H
SSC-H
LIVE
L/D
LYM
NKT
NK
CD56
CD3
ILC
Lin
CD127
ILC2
ILC3
ILC1
CD117
CRTH2
IFNγ
IL-13
IL-17A
IL-22

Figure S6: Unstimulated control for intracellular cytokine staining

Data is shown for unstimulated adult PBMC. Analysis and gating was performed as in Figure S5. Abbreviations used: NK – natural killer cells, NKT – natural killer T -like cells, GrzmB – granzyme B, ILC – innate lymphoid cells. B – counting beads, LD – live/dead, LYM – lymphocytes.

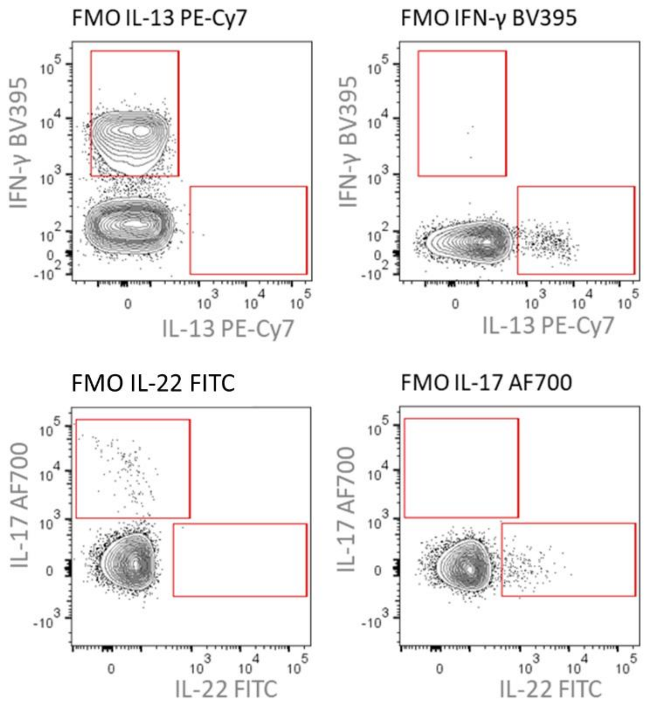

Figure S7. FMO controls for IFN-γ, IL-13, IL-17A and IL-22 staining in lymphocyte populations. Illustrative data is shown for $CD4^+$ T cells in stimulated adult PBMC. The title of each plot indicates the antibody that was excluded from the staining panel for each FMO control. $CD4^+$ T cells were defined within the live, lymphocyte gate as $CD3^+CD56^-CD4^+$ cells.

Figure S8

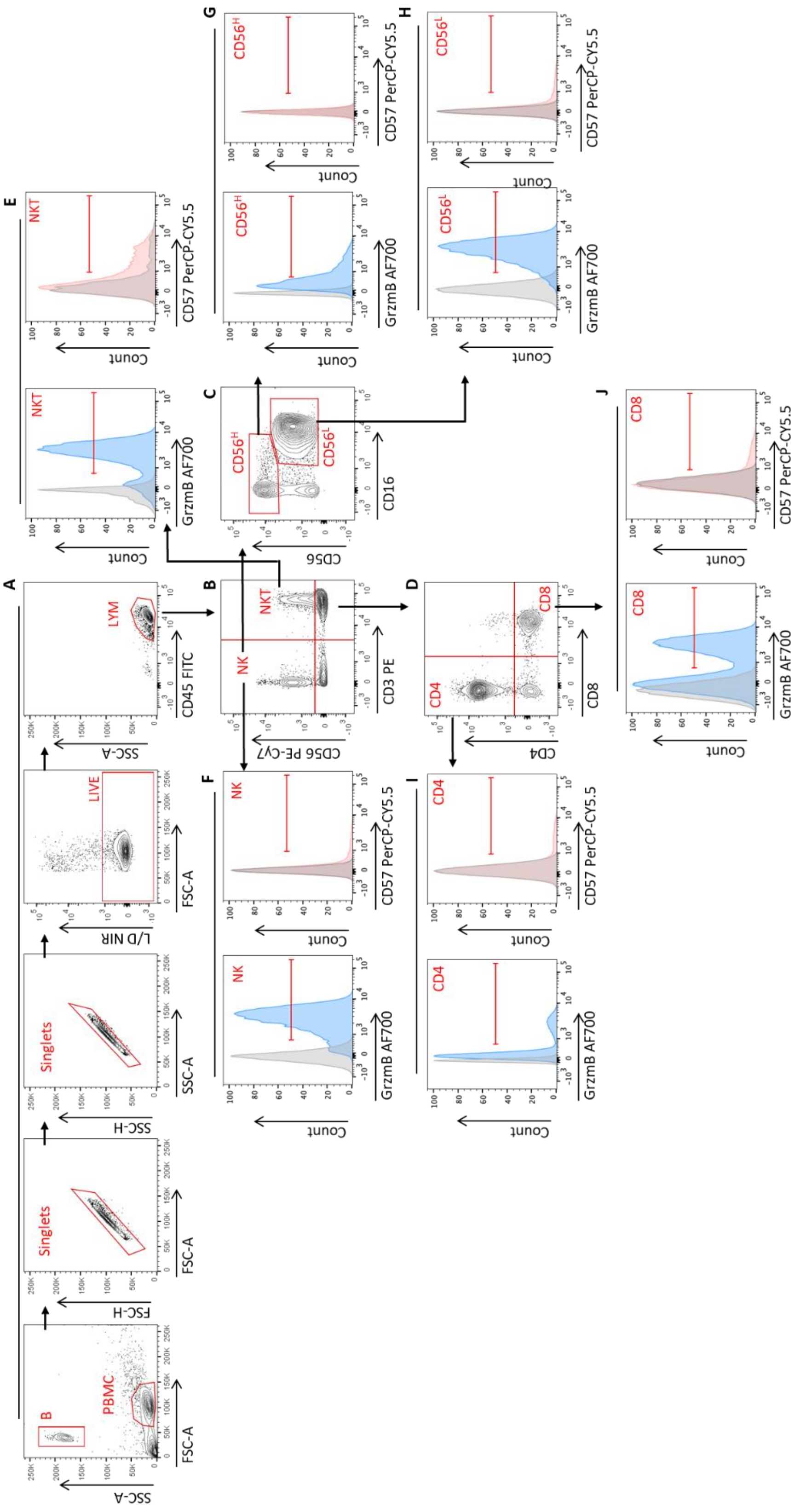
A
B
C
D
E
F
G
H
I
J
PBMC
Singlets
LIVE
LYM
NK
NKT
CD4
CD8
SSC-A
FSC-A
FSC-H
SSC-H
L/D NIR
CD45 FITC
CD3 PE
CD56 PE-CY7
CD16
CD56
GrzmB AF700
CD57 PerCP-CY5.5
Count

Figure S8. Gating strategy for analysis of Granzyme B and CD57 expression in NK, NKT-like, CD4$^+$ and CD8$^+$ T cell populations

(**A**) Cells were delineated using the following gating strategy: FSC/SSC, single, live cells. Lymphocytes were gated using SSC-A and CD45. (**B**) Within the lymphocyte population, NK and NKT-like cells were gated as CD56$^+$CD3$^-$ and CD56$^+$CD3$^+$ cells, respectively. (**C**) NK cells were further subdivided according to their CD56 and CD16 expression. (**D**) T lymphocytes (CD3$^+$CD56$^-$) were segregated into CD4$^+$ or CD8$^+$ T cells. Granzyme B$^+$ and CD57$^+$ cells were identified within (**E**) NKT - like cells, (**F**) NK cells, (**G** and **H**) NK cell subpopulations, (**I**) CD4+ T cells and (**J**) CD8+ T cells. Gates were set according to fluorescence minus one (FMO) controls (grey), with staining for CD57 shown in pink and for granzyme B in blue. *Abbreviations used:* B – counting beads, PBMC – peripheral blood mononuclear cells, LD – live/dead, LYM – Lymphocytes, NK – natural killer cells, NKT – natural killer T -like cells.

Figure S9

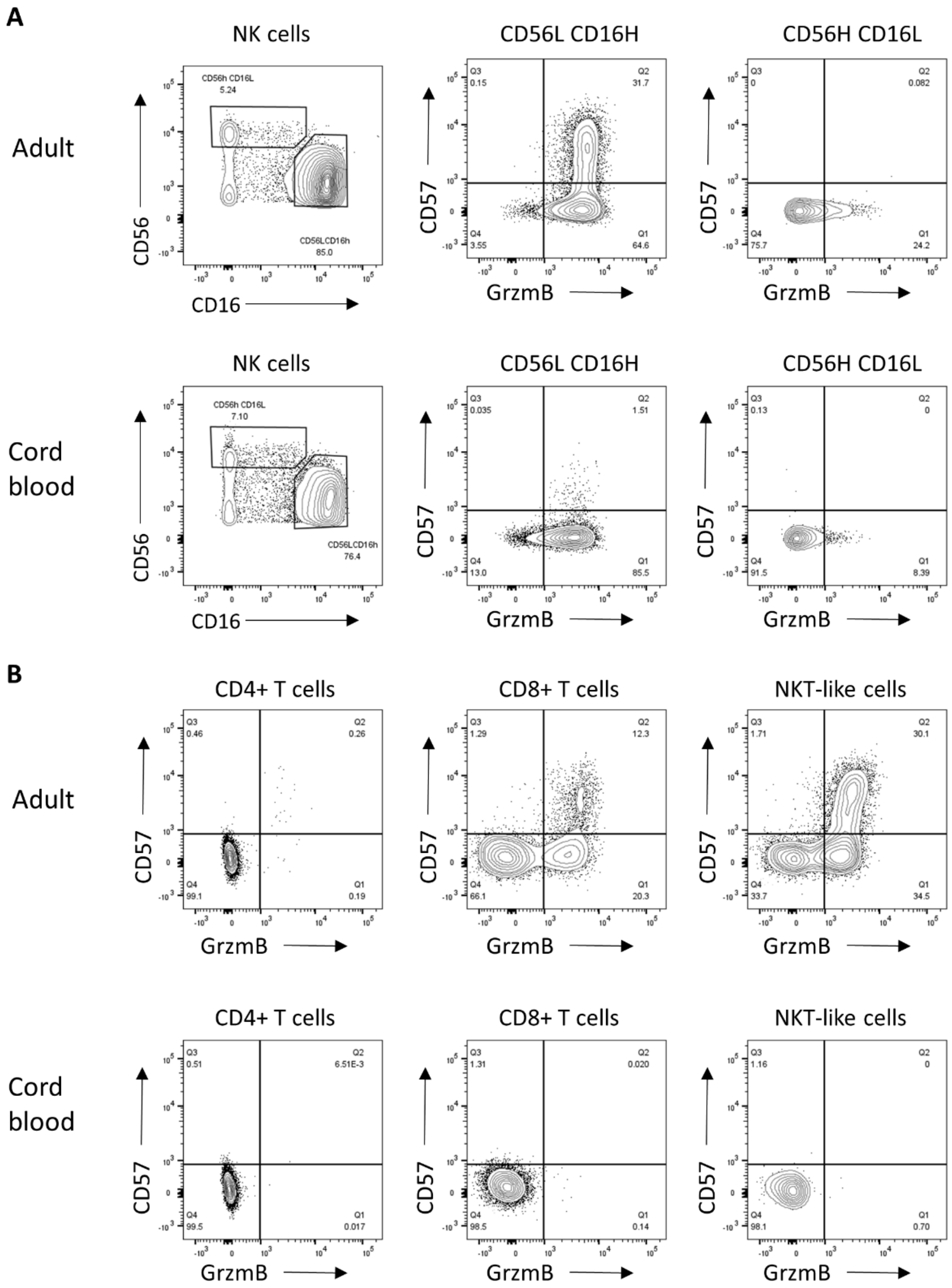

Figure S9. Illustrative data for Granzyme B and CD57 expression in PBMC from adult and cord blood. (**A**) NK cells were defined as live, single, CD45$^+$CD56$^+$CD3$^-$ lymphocytes and sub divided into CD56^{H}CD16^L and CD56^{L}CD16^H populations. (**B**) CD4+ and CD8+ subpopulations were identified within CD3+ T cells and NKT-like cells were defined as CD3+CD56+ lymphocytes. Plots show the expression of granzyme B and CD57 in lymphocytes from adults and cord blood. Data are representative of n=7 donors from each cohort.
